# Network Evolution of Body Plans

**DOI:** 10.1371/journal.pone.0002772

**Published:** 2008-07-23

**Authors:** Koichi Fujimoto, Shuji Ishihara, Kunihiko Kaneko

**Affiliations:** 1 ERATO Complex Systems Biology Project, Japan Science and Technology Agency, Tokyo, Japan; 2 Department of Basic Science, University of Tokyo, Meguro, Tokyo, Japan; 3 Division of Theoretical Biology, National Institute for Basic Biology, Okazaki, Japan; Utrecht University, Netherlands

## Abstract

One of the major goals in evolutionary developmental biology is to understand the relationship between gene regulatory networks and the diverse morphologies and their functionalities. Are the diversities solely triggered by random events, or are they inevitable outcomes of an interplay between evolving gene networks and natural selection? Segmentation in arthropod embryogenesis represents a well-known example of body plan diversity. Striped patterns of gene expression that lead to the future body segments appear simultaneously or sequentially in long and short germ-band development, respectively. Moreover, a combination of both is found in intermediate germ-band development. Regulatory genes relevant for stripe formation are evolutionarily conserved among arthropods, therefore the differences in the observed traits are thought to have originated from how the genes are wired. To reveal the basic differences in the network structure, we have numerically evolved hundreds of gene regulatory networks that produce striped patterns of gene expression. By analyzing the topologies of the generated networks, we show that the characteristics of stripe formation in long and short germ-band development are determined by Feed-Forward Loops (FFLs) and negative Feed-Back Loops (FBLs) respectively, and those of intermediate germ-band development are determined by the interconnections between FFL and negative FBL. Network architectures, gene expression patterns and knockout responses exhibited by the artificially evolved networks agree with those reported in the fly *Drosophila melanogaster* and the beetle *Tribolium castaneum*. For other arthropod species, principal network architectures that remain largely unknown are predicted. Our results suggest that the emergence of the three modes of body segmentation in arthropods is an inherent property of the evolving networks.

## Introduction

Evolutionary diversification of multi-cellular organisms largely depends on body plans, in which complex morphologies develop under the integrated control of multiple genes [1]. The interaction among genes and gene products forms a regulatory network that orchestrates gene expression pattern to specify the morphologies. Mutational modification in gene regulation networks alters gene expression dynamics that provide a basis for morphogenetic diversity. A fundamental key to understanding evolutionary developmental biology is to elucidate how a gene network determines body plan, its diversity, and its potential to evolve [2]–[6]. Here we focus on gene expression patterning in segmented body plans during arthropod embryogenesis as model systems to address this question.

**Figure 1:**
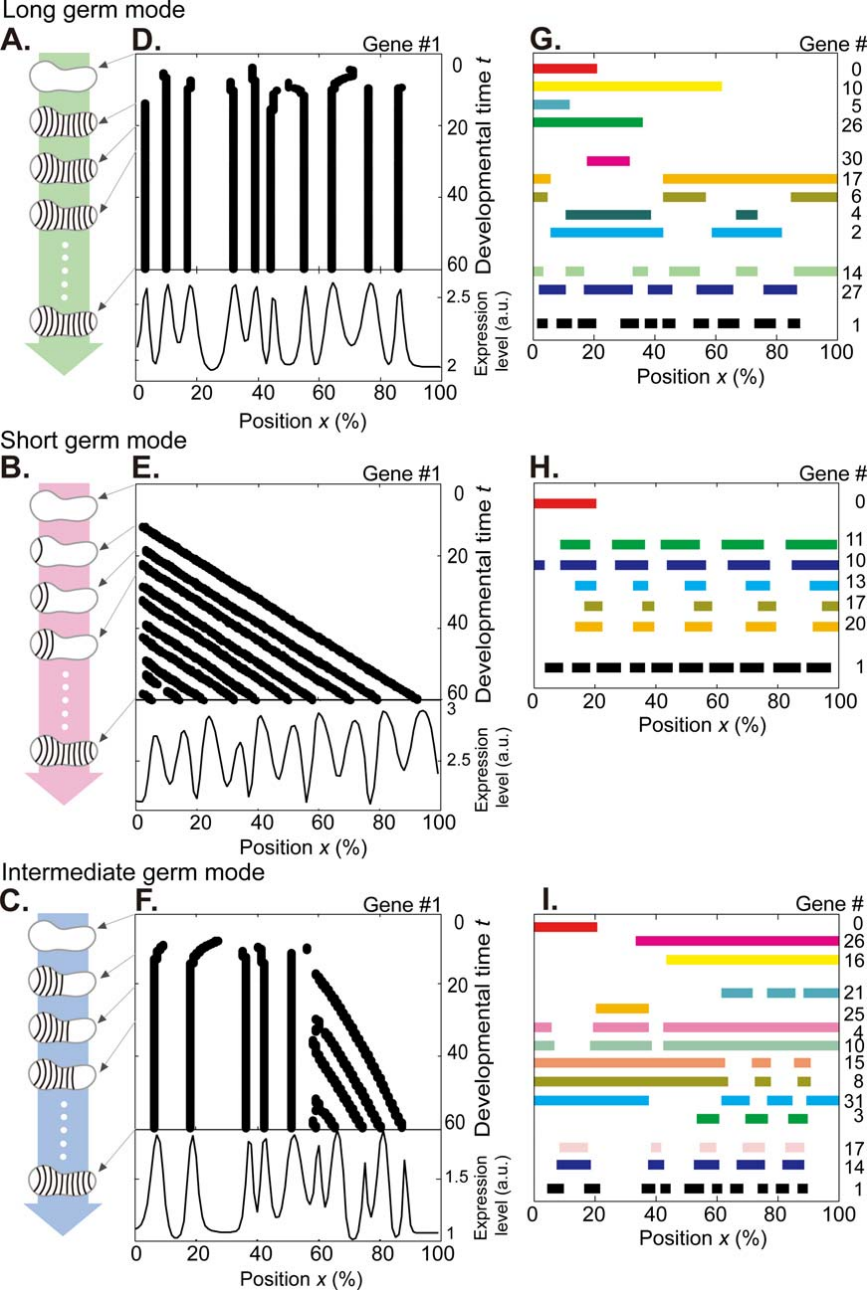
The evolved networks simulate long, short, and intermediate germ-band development. (A–C) Schematic representation for the three modes of embryogenesis. (D–E) Typical spatio-temporal patterns of the gene #1 during development (upper panel) and snapshots of the final established pattern at *t* = 60 (lower panel). The unit *t* is normalized by the timescale of degradation 1/*γ*. Ten segmental stripes appear simultaneously at *t*∼10 in (D), whereas sequentially in (E). In (F), five stripes on the left side first appear simultaneously, and the other five on the right appear sequentially. (G–I) Digitized expression at *t* = 60 for the genes in the core network (Fig. 2A–C) corresponding to (D–F), respectively (See Figure S2 for quantitative expression pattern and Figure S3 for spatio-temporal development). Gene index is indicated on the right. In (G), expression appears in a gradient (genes #0, #10, #5, and #26), a single stripe (#30), two stripes (#2 and #4), five stripes (#27), and ten stripes (#1) respectively. For genes #11, #10, #13, #17, and #20 in (H), the number of stripes that appear sequentially is about half as many stripes for #1. During short germ-band development, pair-rule genes are also expressed sequentially [10] and show half as many stripes for the segment polarity genes [30]. In (I), spatio-temporal dynamics of genes #21, #25, #4, #10, #15, #8, #31, #3, #17, and #14 agree with expression of gap and pair-rule genes in intermediate germ-band insects [12], [13], [31]. a.u.; arbitrary unit.

Arthropod segmentation exhibits three developmental modes of the stripe pattern formation in gene expression that specify the future elementary segments of an adult body [7], [8]. Many of the descendant arthropod species (Fig. 1A; e.g., the fly *Drosophila melanogaster* [9]) follow the ‘long germ-band’ mode of development where stripes appear simultaneously along the anterior-posterior axis. In contrast, ancestral species (Fig. 1B; e.g., the beetle *Tribolium castaneum* [10] and the spider *Cupiennius salei* [11]) exhibit ‘short germ-band’ mode where stripes appear sequentially. A combination of both is found in ‘intermediate germ-band’ mode; anterior stripes appear simultaneously while the remaining posterior stripes appear sequentially (Fig. 1C; e.g., the cricket *Gryllus bimaculatus* [12] and the milkweed bug *Oncopeltus fasciatus* [13]). Conservation of regulatory genes such as gap and pair-rule genes among arthropods indicates that the differences in the stripe formation have originated from architecture of the regulatory network. Comparative studies from species to species have extensively been carried out to reveal differences in spatiotemporal gene expression pattern while knockout responses are studied to decipher a functional role of genes in shaping the morphogenesis [14]–[17].

These observations raise three related problems. First, what is basic difference in network architecture that distinguishes the three modes? Second, how does a distinct network architecture produce spatio-temporal gene expression corresponding to each developmental mode for segmentation? Can the functional role of each network architecture account for observed knockout responses? Third, what type of evolution pressure will favor the selection of each developmental mode? So far the understanding of the evolution of gene regulatory networks remain too fragmentary to answer these questions, due to practical limitations of time scale in experimental approaches.

To address these problems, here we adopt an integrated approach by analyzing structure and function of gene networks, and modeling diversity in striped pattern formation. In order to reveal the basic differences in the network architecture, developmental gene networks are numerically evolved [18]–[23] under selection pressure to form a target number of stripes expressed in a specific gene, which we label #1 without loss of generality (Figure S1; see Methods). We find emergence of three developmental modes to form the stripes. The three modes are characterized by the presence and abundance of Feed-Forward Loops (FFLs), Feed-Back Loops (FBLs), and interconnection between the two types of loops in the gene network. As we will see later, these three modes strikingly agree with long, short, and intermediate germ development in arthropod segmentation respectively, with regard to spatio-temporal gene expression and knockout responses. Furthermore, network architectures composed of FFLs and/or negative FBLs exhibit a trade-off constraint between mutational robustness and developmental speed, which may play a crucial role in the evolution of segmented body plans.

## Results and Discussions

### Three developmental modes in artificial evolution

**Figure 2:**
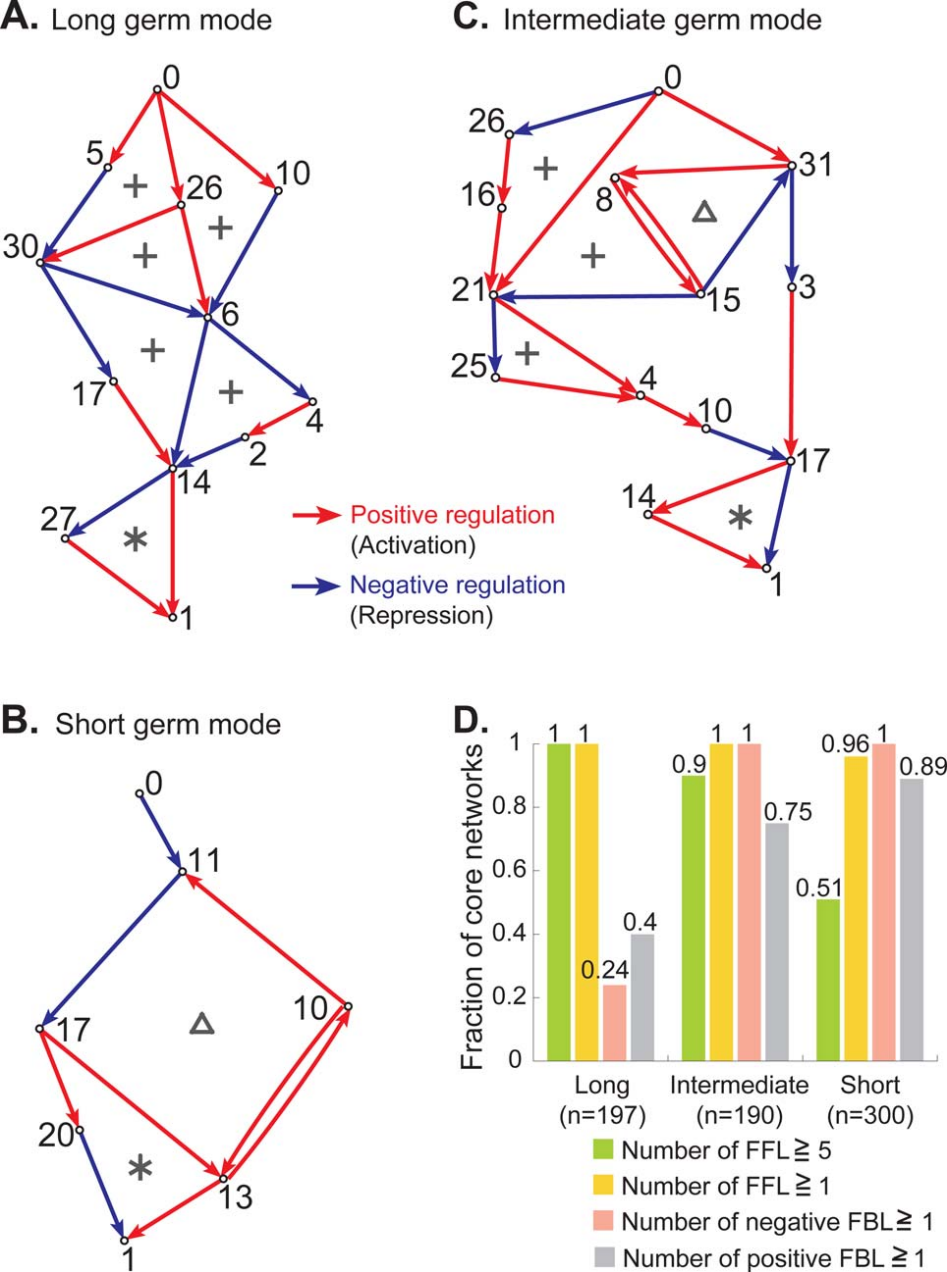
FFL, negative FBL and their interconnections characterize the core network architecture. (A–C) The core networks responsible for generating ten stripes shown in Figure 1D–F, respectively. The number indicates the gene index. (A) An example of a core network having no FBL but multiple FFLs; gene #0 to #30, #0 to #6, #26 to #6, #30 to #14, #6 to #14 (connected in parallel marked by+), and #14 to #1 (connected in series marked by *). (B) A FBL (marked by a triangle Δ) composed of genes #11, #17, #13, and #10 generates stripes sequentially for the respective genes, whereas a FFL connected in series (marked by *) is composed of #17, #13, #20 and #1 (see Fig. 1I for expression pattern of these genes). (C) There exist both FFLs, (indicated by+and *) and a negative FBL (marked by Δ). (D) Statistics of core network architectures represented by the fraction of core networks containing a FFL (light green), five FFLs or more (yellow), a negative FBL (pink) and a positive FBL (gray), respectively (See the distribution for number of FFLs and FBLs in Fig. S6A–B). It can be shown from a theory [42] that the minimum number of FFLs required to generate ten stripes is five.

Within approximately 1000 independent evolutionary trials, we discovered that the selected networks exhibit three basic modes of spatio-temporal gene expression (Figs. 1D–F and S12): simultaneous, sequential, and combinatorial stripe formation. In the mode displayed in Figure 1D, stripes appear almost simultaneously, while in another mode shown in Figure 1E each stripe appears one by one. Figure 1F shows an example of combinatorial formation, where stripes appear simultaneously on the left side but sequentially on the right side. These modes are well known for the spatio-temporal expression of segment polarity genes in the long [9], [24], [25] (Fig. 1A), short [10], [11], [26]–[29] (Fig. 1B), and intermediate [12], [13] (Fig. 1C) germ embryogenesis of arthropods. In addition to simultaneous stripe formation of gene #1, expression of the upstream genes in the network (Fig. 2A) also follows a characteristic pattern observed in long germ insects [9], [24], [25] (Figs. 1G and S2A); a maternal gene in a simple gradient, gap genes in one or two domains, pair-rule genes that form half as many stripes as segment polarity genes – a phenomenon known as ‘double segment periodicity [9], [16]’. Similarly, in networks exhibiting sequential and combinatorial stripe formation, as will be discussed, the expression patterns of the other genes closely follow those reported for short [30] and intermediate [12], [13], [31] germ-band arthropods respectively (Figs. 1H–I, S2B–C, and S3B–C).

### Modularity in artificially evolved networks

In order to find the underlying network properties that give rise to the three distinct developmental modes, we first extracted minimal sub-networks necessary for the striped pattern (Fig. 1D–F) from the evolved networks (Fig. 2A–C; see Methods and other representative examples in Fig. S5). We shall hereafter refer to these as ‘core networks’. Second, the core networks were classified into long, short, and intermediate germ modes according to the exhibited mode of stripe formation as described above (see Methods). Then, for each mode, we investigated the appearances of the two prominent motifs in regulatory networks - FFLs and FBLs [32]–[41]. We have discovered that multiple FFLs (Fig. 2A) are always included in the core networks in the long germ modes while at least one negative FBL (Fig. 2B) is always included in the short germ mode. Figure 2D shows the fraction of core networks that contains FFL and negative and positive FBLs. Multiple occurrences of FFLs in the long germ network (indicated by green bar graph in Fig. 2D) have been observed, while the appearance of at least one negative FBL in the short germ network (indicated by pink in Fig. 2D; Positive FBL is not always included in either long or short germ networks as indicated by gray in Fig. 2D). Both FFL and negative FBL always coexist for the intermediate germ network (Fig. 2C–D).

### Mechanism of striped pattern formation based on FFLs and FBLs

A single FFL functions as a stripe generator [42]–[44] (see Supporting Result S1 for a theoretical analysis). Let us give an example by examining a FFL from gene #0 to #30 in Figure 2A. The FFL lies downstream of maternal factor #0 that is imposed in the form of a simple gradient. Since gene #30 is activated by gene #26 and at the same time repressed by gene #5 depending on the level of #0, expression of #30 appears in a single stripe (Figs. 1D and S2A). The function of FFLs connected in series (marked by * in Fig. 2A) is to double the number of stripes, whereas the function of FFLs connected in parallel (marked by+in Fig. 2A) is to add a stripe [42]. The number of stripes to be added is determined depending on the number of FFLs connected in series or in parallel (Figs. S7 and S8). A negative FBL, on the other hand, functions as a temporal oscillation generator. Short germ development is expected to operate by a mechanism [14], [16] similar to segmentation in vertebrates where oscillations are translated into sequential striped patterns by intercellular interactions [45]–[49]. Genes located either within or directly downstream of a negative FBL are subjected to temporal regulation by the FBL (Fig. S9B), resulting in sequential stripe formation (Fig. S3B). In the intermediate germ mode, genes regulated by a negative FBL (marked by Δ in Fig. 2C) show the sequential stripe formation, whereas genes regulated by FFLs (marked by+in Fig. 2C) show simultaneous stripe formation (Figs. S3C and S9C). These results suggest that parallel connection of FFL and negative FBL organizes the combinatorial stripe formation.

**Figure 3:**
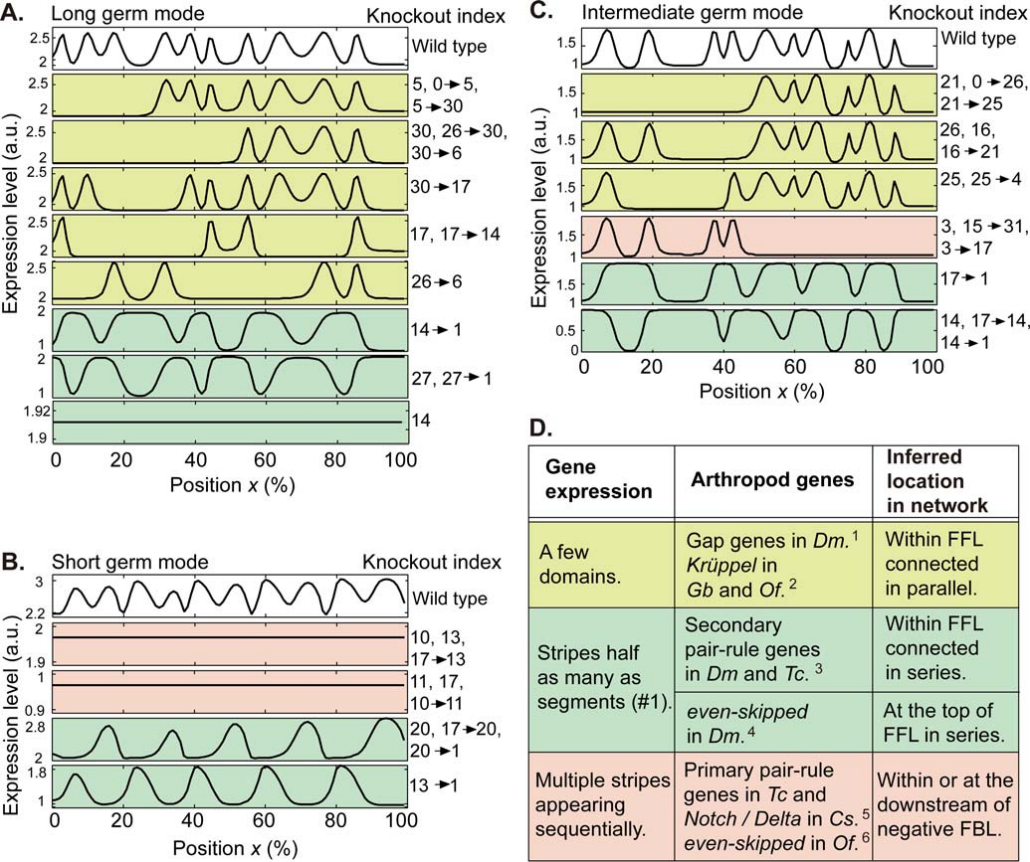
Disruption of FFL and negative FBL induce the characteristic defects in arthropods. (A–C) The knockout index is indicated on the right; e.g., “30→17” and “14” denote removal of a connection between gene #30 and #17 and deletion of gene #14, respectively, whereas “5, 0→5, 5→30” signifies that each response from deleting the gene or connection is the same. The genes and connections belong to either a FFL connected in parallel (colored by yellow green), a FFL connected in series (blue green) or a negative FBL (pink) in the core network (Fig. 2A–C) (see Figure S10 for the spatio-temporal patterns of gene expression). The 1st panel is from the wild type network corresponding to the lower panel in Figure 1D–F. The blue green panels in (A) and (C) show that stripes are fused in pairs to form a single stripe with absence of every other local minima, except for the lowest panel in (A) where a perturbed gene #14 is located at the top of the FFL. (D) Genes indicated by the knockout index (see below) agree with arthropod genes in terms of their spatio-temporal development (Figs. S2 and S3) and the patterns of knockout response (A–C). ^1^Corresponding to gene #30 and #17 in A. ^2^#25 in C. ^3^#27 in A, #20 in B and #14 in C. ^4^#14 in A. ^5^#10, #11, #13 and #17 in B. ^6^#3 in C. *Dm: D. melanogaster*, *Tc: T. castaneum*, *Gb: G. bimaculatus*, *Of: O. fasciatus*, *Cs: C. salei*.

We examined the roles of FFL and FBL by performing ‘knockout experiments’ in all evolved networks (see Methods). The stripes in gene #1 vanish by eliminating a gene or a connection either within or downstream of a FFL or FBL. Perturbations of a FFL connected in parallel (+in Fig. 2A and C) often results in defects confined to a few domains in a long or intermediate germ mode as observed for the gap mutation [12], [13], [50] (yellow green panels in Fig. 3). Disrupting a FFL connected in series (* in Fig. 2A–C) often leads to absence of every other stripes as in the pair-rule mutation [30], [50] (blue green panels in Fig. 3), while disrupting gene at the top of the FFL (e.g., #14 in Fig. 2A) extinguishes all the stripes (the lowest figure in Fig. 3A). By disrupting a negative FBL (( in Fig. 2B–C), stripes that are formed sequentially are extinguished completely in short and intermediate germ modes (pink panels in Fig. 3).

The function of positive FBL sharpens striped pattern through interaction with a FFL [51] and amplifies temporal oscillation through the interaction with a negative FBL. However each role of positive FBL can be substituted by FFL and negative FBL, respectively, by tuning up parameter values in the FFL and the negative FBL through evolution. Thus a positive FBL is not necessary module (Fig. 2D). These results indicate that FFL and negative FBL are elementary modules responsible for the three characteristic modes of development.

### Network architecture in arthropod segmentation

In contrast to detailed models for a specific species [52], [53], our aim is to capture general consequence of evolution of gene expression dynamics that hold over a large number of both artificial and arthropod networks. All the evolved network models we examined were exactly classified into three modes, sequential, simultaneous or combinatorial formation, respectively. We identified necessary network module for each mode (Fig. 2D) and confirmed its function for the stripe formation (Fig. 3A–C and Result S1). Characteristics in spatiotemporal gene expression pattern and the network structure are summarized in Table 1. These three modes in our models agree rather well with the short, long, and intermediate modes in arthropods.

**Table 1:** Summary of the three developmental modes in our models.

**Developmental mode**	Long germ	Short germ	Intermediate germ
**Stripe formation** 1	Simultaneous	Sequential	Combinatorial
**Network module** 2	Multiple FFLs	A negative FBL	FFL and negative FBL
**Variety of expression patterns** 3	Necessary	Not necessary	
**Variety of knockout responses** 4	Necessary	Not necessary	
**Mutation rate** 5	Lower	Higher	
**Developmental speed** 6	Slower	Faster	

1 Fig. 1A–F.

2 Fig. 2.

3 Figs. 1G–I, S2 and S7.

4 Fig. 3.

5 Fig. 4C.

6 Fig. 4E.

Strikingly, besides the above correspondence in segmentation modes, we almost always find genes that qualitatively agree with arthropod genes in terms of how, where and in what order these genes are being expressed (Figs. 1G–I, S2, and S3). Moreover, when these genes are deleted from the network and compared with the respective knockout mutants in real arthropods, the altered expression patterns of gene #1 (Fig. 3A–C) and the segment polarity genes exhibit remarkable similarities (Fig. 3D). By focusing on the function of FFL and negative FBL, where the networks modules are located in the arthropod gene regulatory networks and how the arthropod genes are wired are straightforwardly inferred from mapping them to the corresponding genes in the artificial networks.

#### Gap genes

As shown in Fig. 1G and I, several genes express in a few domains generated by FFL connected in parallel (see also Fig. S7A–C, and 2nd figure in Fig. S2A and C). Whenever one of the genes is disrupted, a defect of striped pattern is produced locally for a corresponding domain (yellow green panels in Fig. 3). For example, such response is shown in the knock-out of gene #30 and #17 in Fig. 3A, and #25 in Fig. 3C. Indeed, these types of expression pattern in wild type and local defect of stripes induced in segmentation gene are known as roles of gap genes in a long germ insect *D. melanogaster* [49], and a gap gene *Krüppel* in intermediate germ insects *G. bimaculatus* [12] and *O. fasciatus* [13]. Even though detailed knowledge on the gene network for them is not yet available, we infer here that the arthropod genes should be located within a FFL connected in parallel, as in #30 and 17 in Fig. 2A, and #25 in Fig. 2C.

#### Pair-rule genes

In our models, several genes exhibit the double segmental periodicity generated by FFL connected in series where the stripe number is as half as that of segmentation gene #1 (Fig. 1G–I). Disrupting one of the genes located within the FFL always leads to absence of every other stripe with deletion of odd- or even-numbered stripe (blue green panels in Fig. 3B) or fusion of each pair of two stripes (Fig. 3A and C), while disrupting a gene at the top of the FFL extinguishes the stripes (the lowest panel in Fig. 3A). For example, the former response appears by the knock-out of gene #27 in Fig. 3A, #20 in Fig. 3B and #14 in Fig. 3C, whereas the latter by the knockout of gene #14 in Fig. 3A. Both the double segment periodicity and the mutant phenotype emerge as a result of the FFL connected in series (Fig. S7D). Indeed, the double segment periodicity is widely observed in arthropod pair-rule gene expression [9], [10], [26], [29], [30], [54] . Disrupting the secondary pair-rule genes [9] in *D. melanogaster* and *T. castaneum* (short germ) leads to absence of every other stripes in segment polarity gene expression with the deletion [30], [50] or the fusion [50], [55] of every other stripe, while null mutation of the primary pair-rule gene *even-skipped* in *D. melanogaster* extinguishes the segments [56]. Thus the arthropod secondary and primary pair-rule genes are expected within a FFL connected in series (e.g, #27 in Fig. 2A, #20 in Fig. 2B and #14 in Fig. 2C), and at the top of the FFL (#14 in Fig. 2A), respectively.

#### Genes which express striped pattern sequentially

In short germ network models, several genes in a negative FBL express striped pattern sequentially from the anterior to posterior end while disrupting one of the genes always extinguishes almost all the stripes (e.g., gene #10, #11, #13 and #17 in Figs. S3B and 3B). In intermediate germ network models, a gene subjected to a negative FBL expresses striped pattern sequentially around posterior end while disrupting the gene extinguishes the stripes at the corresponding domain in the wild type (#3 in Figs. S3C and 3C). Moreover, striped pattern among genes in the FBL is partially overlapped, irrespective of the developmental modes (e.g., Fig. S2B). We have found such partial overlap only when the genes are located in a negative FBL (Δ in Fig. 2B). Indeed, these types of spatio-temporal expression and knockout responses were reported in primary pair-rule genes in *T. castaneum* [30], [57], *Notch/Delta* in *C. salei* (short germ) [11], and *even-skipped* in *O. fasciatus* [31]. Thus these arthropod genes are expected to be located either within (e.g., #10, #11, #13 and #17 in Fig. 2B) or at the downstream of a FBL (#3 in Fig. 2C).

Abundance and interconnection of FFLs in accordance with the above predictions are well documented in *D. melanogaster* [42], [58]. For example, existence of FFL composed of primary and secondary pair-rule genes and segment polarity gene was reported (Fig. 5 in ref. [55]). For *T. castaneum* [30], genetic studies suggest that the primary and secondary pair-rule genes are located within a negative FBL and a FFL connected in series as shown in Figure 2B. We infer that the negative FBL and FFL are responsible modules for forming stripes sequentially and double segmental periodicity, respectively. Spatio-temporal expression and knockout response of evolutionarily conserved genes such as *even-skipped* may differ dramatically from species to species [8], [16], [17], [59]. The above results exemplify the necessary rewiring of FFLs and/or negative FBLs that must have taken place during the arthropod evolution.

### Network modularity and the robustness in developmental evolution

We now discuss implications of the network architectures derived from our models to each developmental mode and evolutionary process. The hierarchical structure of FFLs add or double stripes in order to form multiple stripes in all long germ core networks; a gene expressed in a simple gradient (#10 and #26 in Figs. 1G, S2A and 2A, and Result S1) is followed by genes that are expressed in one or two stripes (#30 and #6). They are further connected to genes appearing in many more stripes (#14 and #1). The knockout response varies depending on the exact position of the disturbed FFL in the core network (Fig. 3A). On the other hand, variations in striped pattern are only occasionally observed in short germ networks. The majority of the mutant networks show no changes in the number of stripes while a very small fraction of them fails to form stripes all together (Fig. 3B). Hence, a hierarchy of FFLs and a variety of knockout responses are necessary features of the long germ development. In contrast, for the short term development, there is no such hierarchy and consequently, no strict necessity in variety of knockout response.

**Figure 4:**
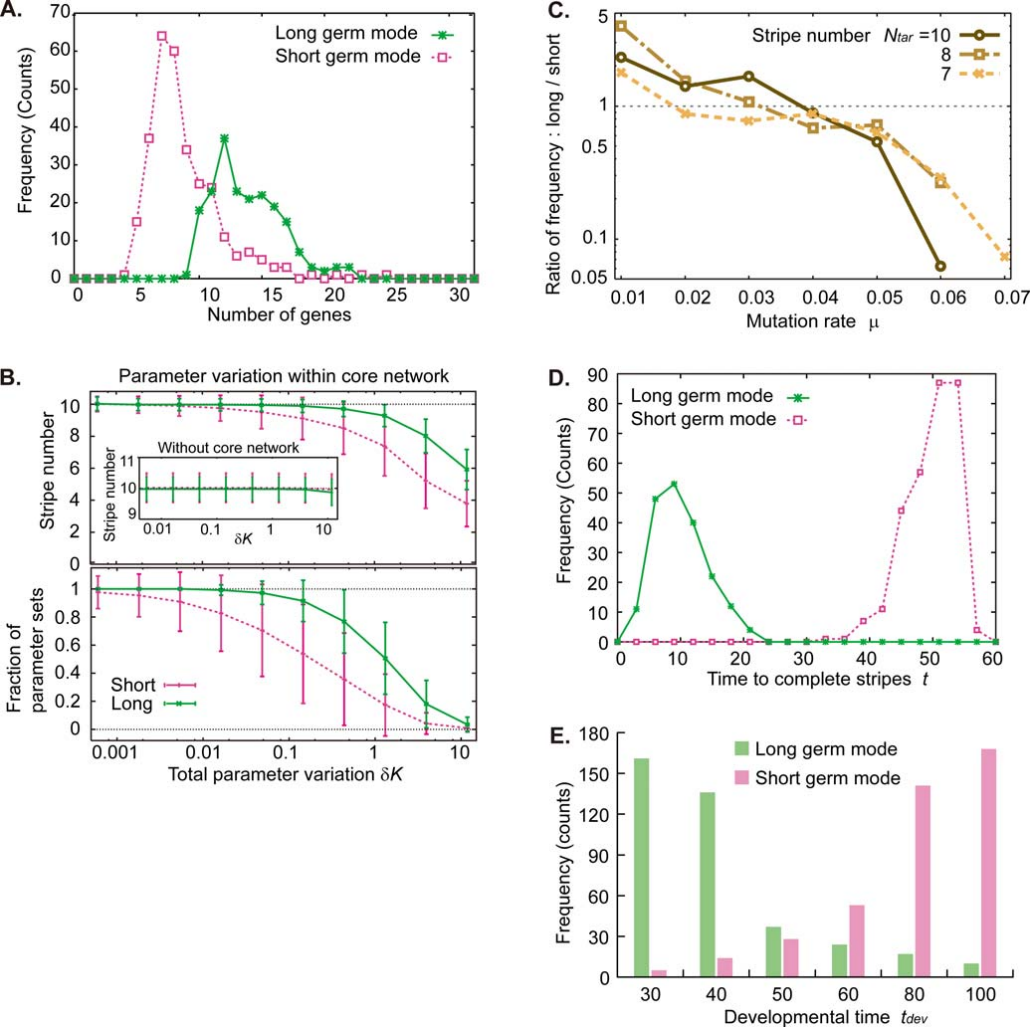
Trade-offs between long and short germ modes in development (A, B, D) and evolution (C, E). (A) Frequency distribution of the number of genes is plotted for core networks of long (green) and short (pink) germ modes. (B) Robustness of stripe number expressed in gene #1 to parameter variation. By generating an ensemble of systems subjected to parameter change from a given reference system, the fraction of such systems that maintain all the stripes of the original system (upper figure) and the average stripe number among the ensemble (lower figure) are plotted as a function of the total parameter variation *δK* (See Methods). The variations are introduced into the threshold parameters *K_j→i_* in the paths within a core network, while variation into connections without core networks hardly induces stripe defect (inset). (C) When the networks are evolved under different mutation rate *μ* the ratio between the frequency of long germ mode and that of short germ mode is plotted against *μ* (See the absolute frequency in Fig. S11). (D) Developmental time required for the stripe formation is shorter for the long germ networks (green) than the short germ networks (pink). For each evolved network, the time it takes to complete formation of the target number of stripes for gene #1 was measured. The distributions in (A) and (D) and error bar in (B) are computed from an ensemble of networks also used to obtain Figure 2D. (E) When the networks are evolved under different developmental time constraints, frequencies of appearance of long and short germ modes are plotted against the length of development *t_dev_* (see Methods). Target stripe number *N_tar_* = 10 and mutation rate *μ* = 0.05.

The susceptibility to network perturbation (Fig. 3) is known as robustness of the network [21]–[23], [52], [60]–[63]. The small size of the core network (Figs. 4A and S6C–D) implies less chance for the dynamics to be disrupted by mutation. Of course how a certain gene regulatory network works depends not only on the topology but also on the parameters of gene regulation *K_j→i_*. As can be inferred from the earlier studies of FFLs [42], they work at a certain range of parameters. Here, we have found that the evolved network has robustness against parameter variation in *K_j→i_* under fixed network topology. In contrast to perturbation on the topology, the parameter robustness is stronger for long-germ networks than short-germ networks (Fig. 4B; see Figure S12 also for robustness to noisy perturbation in development).

Mutational robustness in evolution could be described by a trade-off between two features of the robustness to network topology and parameters. Comparing the networks evolved under different mutation rate *μ* (i.e. the probability of genetic change introduced in a network element per evolutionary generation; see Methods), short germ networks appear more frequently at a higher mutation rate *μ* (Fig. 4C). On the other hand, simultaneous expressions of stripes take a shorter developmental time than sequential ones (Fig. 4D). Hence, long germ modes appear more frequently under a selective pressure for rapid development (Fig. 4E). Transitions between short and long germ-band development occurred during evolution of arthropods [7], [8], [14]–[16], [49]. This trade-off between the mutational robustness and developmental speed may provide an evolutionary transition from short to long germ mode.

### Future problems

Even though we have confirmed correspondence between our models and arthropod in segmentation, there remain some problems that have to be clarified in future: First, peak position of striped pattern in a gene expression is less homogeneous in many of long germ network models (Figs. 1D and S5A) compared with those observed in arthropod. Here, detailed peak position can depend more sensitively on the parameters in development. Even under fixed network topology, the heterogeneity in the peak-to-peak distance in the model was reduced by tuning the parameter values through a suitable selection pressure (Figure S14). Second, we have not so far found any short germ network model with the two roles of gap genes on wild type expression and knockout response described above while they were well documented in *T. castaneum* [64]–[67]. It might be related to embryo growth around posterior side [15] that was not considered here. Third, the positive FBLs is not a necessary module in our models, while it is necessary to quantitatively reproduce spatial and temporal expression of gap [53] and segment polarity [62] genes in *D. melanogaster*. The present study focuses on rather qualitative aspects of stripe formation and knockout responses to capture a unifying view among diverse striped patterns. The relationship between FFLs and positive FBL will be addressed in evolution of both quantitative and qualitative information in spatial pattern. Last but not the least, evolutionary transition process among the three developmental modes is an important issue to be studied along the line of our study.

### Conclusion

Our aim here is to elucidate a unifying mechanism behind diverse processes across species. We derive four predictions regarding the network architectures of arthropod segmentation. First, in all long germ arthropods, gene regulatory networks should always exhibit a hierarchical structure composed of multiple FFLs, and the striped pattern of mutants should exhibit a variety of forms. The short germ arthropods, on the other hand, should not necessarily show such a hierarchical structure or a variety in knockout responses. The second is the absolute necessity of a negative FBL for short germ arthropods. Third, an interconnection of FFL and negative FBL is essential for intermediate germ development. And lastly, the double segment periodicity is a signature of spatial organization by serially connected FFLs. For *T. castaneum*, the negative FBL and FFL composed of pair-rule genes [30] should form stripes sequentially and double segmental periodicity, respectively. Although the above predictions should be carefully tested, the overall agreement between our highly abstract model and the well-studied arthropods indicates that the appearance of long, short, and intermediate germ-band development are not by chance but rather by necessity [18], [68], [69] in the evolution of segmented body plans.

Note added in Proof: In a recent publication [70], evolution of gene network for segmentation is also studied. In particular by focusing on short germ development, they implemented embryo growth at the posterior end to understand ceasing temporal oscillation, known as “clock and wave front” model [71]. They found the mechanism through the interaction of time periodic gene expression and morphogen gradient that moves along with posterior growth. In the present paper, the growth was not concerned and ceasing oscillation rarely appears in short germ mode (Fig. S5B). In contrast, we here have identified for the first time responsible network modules for long and intermediate germ modes as well as short germ mode, and clarified these function. From the analysis of the network architecture, we have explained not only the characteristics of each mode but also many of knockout phenotypes, and predicted arthropod gene network topology.

## Methods

### Gene network model for development

Gene expression is governed by a regulatory network [21], [53], in which a single node indicates a single gene, and a connection with an arrow indicates a regulation of a downstream gene #i by an upstream gene #j (see Fig. 2A–C). Architecture of the network is represented by a connection matrix *c_j→i_* where *c_j→i_* = 1, −1 and 0 indicate positive (a red arrow in Fig. 2A–C), negative (a blue arrow), and no regulation, respectively. Expression level of gene #i is represented by the concentration of its product, e.g., protein, *P_i_*. The dynamics of the gene expression obeys
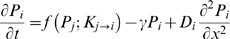
(1)where *γ* is the degradation rate constant, *D_i_* is the diffusion coefficient of the gene product #i, and *x* is the position along the anterior-posterior axis in the embryo. The regulation mediated by gene #j follows a Hill equation 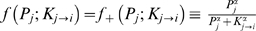 for a positive regulation (*c_j→i_* = 1) or 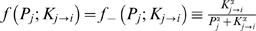 for a negative regulation (*c_j→i_* = −1). Here, *K_j→i_* is a threshold and *α* is a Hill coefficient. When two genes regulate a gene, combinatorial regulation is introduced (See Supporting Methods S1). For the developmental process, equation 1 is numerically integrated starting from uniform initial concentrations in space for all gene products (*P_i_*(*x*) = 0.1) except for *P_0_*(*x*). Gene #0 is the maternal factor, which has no regulator. It is synthesized at and diffuses from one pole of the embryo to establish a simple gradient of the form *P_0_*(*x*)* = A*exp(−*x*/*λ*) at *t* = 0 (See Methods S1). The unit of time *t* is normalized by the timescale of degradation, 1/*γ*. Other parameters are: *α* = 2, *γ* = 1, *A* = 4, and *λ/L* = 0.14 where size of an embryo is given by *L* = 100. 100 cells are arranged in the anterior-posterior direction.

### Evolution of gene network

A single generation of the evolutionary dynamics is composed of (i) mutation, (ii) development, and (iii) selection (Fig. S1A). (i) From all *N_s_* networks selected at the previous generation, *N_m_* offspring networks are generated by changing the following network elements where *N_s_* and *N_m_* denote the number of selected and offspring networks, respectively: the connection matrix *c_j→i_*, the threshold value of each connection *K_j→i_*, and the diffusion constant for each product *D_i_* in equation 1. Probability that mutation is introduced in each one of the above elements is defined by the mutation rate *μ*. The total number of networks in the present generation is *N_s_N_m_*. (ii) For development, we carried out numerical calculations of equation 1 from *t* = 0 to *t* = *t_dev_*, and examined the number of stripes in spatial expression pattern of gene #1 for *N_s_N_m_* network. (iii) The closeness between the number of stripes for gene #1 at *t* = *t_dev_* and a target number *N_tar_* was chosen as a fitness function. Neither detailed position of the stripes, transient behavior of gene #1, nor expression of the other genes is accounted for the fitness. *N_s_* highest networks in the fitness were selected from the *N_s_N_m_* networks. These steps complete one generation, and the same procedures are repeated for 2000 generations as a single evolutionary experiment (see evolution of stripe number in Fig. S1B). All elements of the initial networks are set completely at random with no account of prior knowledge of arthropods. We repeated the artificial evolution several hundred times for any given evolutionary condition defined by *N_tar_* and *μ* (*N_tar_* = 10 except for Fig. 4C). For the present work, we choose *N_s_* = 10, *N_m_* = 10, and *t_dev_* = 60 except for Figure 4E. (See Methods S1 for further information.)

### Classification of developmental modes

When the time required to complete stripes of gene #1 expression is less than a certain threshold *t_dev_*/2 and all the stripes appear without temporal oscillations in development, the network is classified into a long germ mode. When the time is longer than the threshold and each stripe appears one by one as they oscillate, the network is counted as a short germ mode (See the temporal oscillations in Fig. S9). When a part of the stripes appears within *t_dev_*/2 and without oscillation, while the remaining stripes appear one by one together with oscillations, the network is classified into an intermediate germ mode. Since the time is different between long and short germ modes (Fig. 4D), the classification is little affected by the choice of the threshold.

### Extraction of core networks

We systematically eliminated regulatory connections in the gene networks keeping the number of stripes expressed for gene #1 at *t* = *t_dev_* (See Fig. S4). If the stripes remain unperturbed by the tentative removal of a connection, the connection is eliminated from the network. This process is repeated until no further elimination is possible. The extraction yields a unique network irrespective of the order of elimination for the majority of the networks.

### Network modules

When a regulatory connection from a node is looped back to regulate itself via other nodes, it is called a Feed-Back Loop (FBL). A direct auto-regulation is not counted as a FBL. Influence of the feedback regulation in total is classified into negative FBL (see examples marked by Δ in Fig. 2B–C) and positive FBL (e.g., a FBL composed of genes #10 and #13 in Fig. 2B), respectively. When a node regulates another node by two different connections, either directly or indirectly, the sub-network composed of the nodes and their connections are called a Feed-Forward Loop (FFL: e.g., * and+in Fig. 2A–C). The FFL is a loop as structure, but not as a directed network. Here we follow the use of this term by Alon et al., which is widely adopted [58]. Unlike their definition [58], it should be noted that the number of genes within a module is not constrained to three in the present work. This is because it can be analytically shown that the ability of a FFL to form a stripe does not depend on the number of constituent nodes (Fig. S7). We counted the number of FFLs and negative and positive FBLs in each core network (Figs. 2D and S6A–B; See also Fig. S13 for the demonstration of modules extracted from core networks shown in Fig. 2A–C). The number of the core networks used to derive the statistics is 197 for the long germ, 300 for the short germ, and 190 for the intermediate germ networks. When all regulatory pathways from “input” gene #0 to “output” gene #1 pass through a network module (e.g., FFL marked by * in Fig. 2A–C), we defined it as connection in series. When some pathways from gene #0 to #1 go through a module and the others do not (e.g., FFLs marked by+in Fig. 2A and C, and a negative FBL marked by Δ in Fig. 2C), we defined it as connection in parallel.

### Knockout experiments

Mutant networks are generated by eliminating a connection or a node from the original network. Elimination of a node is implemented by setting the expression level of the corresponding gene product *P_i_* to 0 throughout development. Likewise, a regulatory connection is eliminated by setting *c_j→i_* to 0. Upon completion of the mutant network development, spatial expression pattern of gene #1 is measured.

### Parameter robustness of striped pattern

Maintenance of the stripe number against variation of parameters is investigated. We chose a reference system that exhibits formation of given stripe number, and perturbed the system by randomly modifying its threshold value of gene regulation *K_j→i_* while preserving the network topology. An ensemble of a thousand altered systems was thus generated. Each alternation of the reference system was characterized by the total parameter variation *δK*, which was introduced [60] as: 
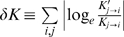
, where *K_j→i_*′ is parameter in the altered system. Development of the altered system was subjected to reaction-diffusion process. Following the developmental process, we measured the fraction of the altered systems that maintain the same stripe number as the original reference system.

## Supporting Information

Methods S1 (0.13 MB PDF)

Result S1 Theoretical Analyses of Gene Regulation in a Long Germ Network.
(0.06 MB PDF)

Figure S1 Evolution of the Striped Pattern. (A) Schematic representation of our numerical evolution. A generation is composed of mutation, development, and selection. (B) The average number of stripes for the selected top *N_s_* = 10 embryos, plotted against generation steps. Nine evolutionary trials are shown. The number of the stripes reaches the target *N_tar_* = 10 after several hundred generations, and it is thereafter sustained. The networks selected at 2000th generation after the evolutionary trials plotted in green, pink, and blue exhibit development shown in Fig. 1D–F, respectively.
(0.29 MB PDF)

Figure S2 Quantitative Representation of the Final Spatial Profiles of Upstream Gene Expression. (A–C) The final expression levels of genes in the core network (Fig. 2A–C) plotted as a function of position. The Y-axis is in a logarithmic scale for B and C. The color representation for the gene index corresponds to that of the digitized plot of gene expression in Fig. 1G–I, respectively. See the spatio-temporal expression for the genes represented by bold lines in Fig. S3). Gene #0 shown in the uppermost panels in A–C forms a spatial gradient as observed in the maternal genes in arthropods [27], [28], [72], [73]. Gene expression shown in the second panel for A and C is confined to a few domains as in the gap genes [12], [13], [25]. Gene expression patterns shown in the 3rd panel of A and 4th panel of C, and those in B except gene #0 show stripes half as many as those in gene #1, consistent with the pair-rule and segment polarity gene expressions [9], [24], [30], [54].
(0.12 MB PDF)

Figure S3 The Developmental Time-course of the Upstream Gene Expression. Local maxima of gene expression are plotted against developmental time, where colors indicate the corresponding genes shown in Figure 1G–I, respectively. (A) Genes begin to be expressed almost simultaneously at 0<*t*<15 in the order of regulatory pathway (Fig. 2A), i.e., gene #30, #14, #27 and #1. After this transient, the positions of expressed genes are maintained over the subsequent development. (B) Genes organized by a FBL (marked by Δ in Fig. 2B) are expressed sequentially to form stripes in #11 and #20, as in pair-rule genes and *Notch/Delta* related genes in short germ arthropod [10], [74], [75]. Next, FFL connected in series (* in Fig. 2B) double the stripes into gene #1. (C) First, a FFL (composed of gene #0, #26, #16 and #21 in Fig. 2C) generates a stripe in gene #25. Another FFL (composed of #21, #25 and #4) doubles the stripe in gene #4, resulting simultaneous appearance of two stripes in #14 at *x*<50. On the other hand, the stripes of gene #3 are formed sequentially by a FBL (marked by Δ in Fig. 2C), resulting sequential appearance of three stripes in #14 at *x*>50. Serially connected FFL (marked by * in Fig. 2C) doubles the stripes of gene #14 and output to gene #1. The posterior side shows coherent development of gene #3 and #1 as in *even-skipped* and segment polarity gene expressions in *O. fasciatus* [31], whereas the anterior side shows coherent pattern of gene #25 and #1 as in *Krüppel* and segment polarity gene expressions in *G. Bimaculatus* [12] and *O. fasciatus* [13]. Knockout experiments confirmed the functionalities of the corresponding network modules (Figs. 3 and S10).
(0.38 MB PDF)

Figure S4 The Core Networks Accurately Reproduce the Developmental Dynamics of the Original Networks. (A–C) The original gene regulatory networks from which the core networks shown in Figure 2A–C were derived. (D–F) Expression pattern of gene #1 generated by the original network (a right blue dashed line) and by the extracted core network (a black solid line, the same as the lower panel in Figure 1D–F, respectively). Not only the number but also the pattern profiles are almost identical in the original and the core networks. The basal level, however, may be shifted as shown in E.
(0.14 MB PDF)

Figure S5 Evolved Networks Show Distinct Network Motifs. Representative examples classified to the three modes are displayed by spatio-temporal expression of gene #1 (lower column) and the corresponding core networks (upper column). (A) Simultaneous gene expression and multiple FFLs in long germ networks. (B) Sequential expression and the presence of a FBL. (C) Combination of simultaneous and sequential expression, and coexistence of FFL and FBL are always observed.
(2.14 MB PDF)

Figure S6 Statistics of Core Network Topologies. (A–C) Frequency distribution of the number of FFLs (A), negative and positive FBLs (B), and regulation, i.e., connections (C) are plotted for core networks of long (green), short (pink), and intermediate (blue) germ modes. The frequency is computed from an ensemble of the core networks used to calculate the distribution in Figure 2D. (D) The average number of genes (black; Fig. 4A) and connections (red; (C)) are plotted for each mode. Error bars indicate the standard deviation. The number of connections as well as the number of genes in the long germ networks is larger than those in the short germ networks. This is because only a single negative FBL is sufficient for the stripe formation in a short germ network to operate, whereas long germ networks require multiple FFLs (Figs. 2D and 3A–B).
(0.11 MB PDF)

Figure S7 Analyses of Gene Regulation in a Long Germ Network. (A) An isolated FFL, (B,C) FFLs connected in parallel (e.g., marked by+in Fig. 2A) and (D) FFLs connected in series (e.g., * in Fig. 2A). Solid lines represent integrated Gene Regulation Functions (GRFs) for genes #30 (A), #6 (B), #14 (C), and #1 (D) plotted against the expression level of gene #0. The other lines are obtained from decomposed GRF (see Result S1). The colors of lines correspond to the colors of arrows in the decomposed pathways shown in each figure. The integrated GRF forms a local maximum in A, two local minima in B, five local minima in C, and ten local maxima in D. Multiple stripes are generated accordingly (Figs. 1G, S2A and S8). *K_0→5_* = 4, *K_5→30_* = 5×10^−2^, *K_0→26_* = 0.7, *K_26→30_* = 0.6, *K_0→10_* = 6×10^−2^, *K_10→6_* = 3×10^−2^, *K_30→6_* = 1.2×10^−2^, *K_26→6_* = 1.4×10^−2^, *K_30→17_* = 1.5×10^−2^, *K_17→14_* = 1.4×10^−2^, *K_6→14_* = 4.7×10^−2^, *K_6→4_* = 3.6×10^−2^, *K_4→2_* = 2.5×10^−2^, *K_2→14_* = 2.3×10^−2^, *K_14→1_* = 0.1, *K_14→27_* = 0.23, *K_27→1_* = 0.26.
(0.16 MB PDF)

Figure S8 Comparison of GRFs Constructed from a Readout of Spatial Pattern and Integrated GRFs. Dotted lines indicate GRFs of gene (A) #30, (B) #6, (C) #4, and (D) #1 constructed from their spatial patterns shown in Figures 2D and S2A, respectively. Solid lines in A–D indicate corresponding integrated GRFs shown in Figure S7A–D, respectively. They show good agreements with each other. The basal level, however, may be shifted as shown in D.
(0.10 MB PDF)

Figure S9 Dynamics of Gene Expression during Development. Expression level of gene #1 is plotted against the developmental time, corresponding to Figure 1D–F, respectively. The spatial positions where the expression level was measured are indicated at the upper right-hand-side corner. (A) After transient expression around *t* = 8, the levels at all positions are maintained without oscillations. (B) After the transient around *t* = 8, temporal oscillations appear sequentially, whereby the timing of the appearance is in the order from the left to the right side of an embryo. (C) The level only at *x* = 60 and 80 shows oscillation in a sequential order.
(0.12 MB PDF)

Figure S10 Developmental Time-course of Mutated Networks. Panels show the expression dynamics of gene #1 organized by a mutant network denoted on the right side. Local maxima of the expression level of gene #1 are plotted at each developmental time *t*. They are in the same conditions as in Figure 3A–C, respectively, except for the cases where the stripes are extinguished throughout the development (the lowest panel in Fig. 3A and pink panels in Fig. 3B).
(0.38 MB PDF)

Figure S11 Short Germ Modes Appear More Frequently in Evolution When the Mutation Rate is High. Approximately four hundred independent evolutionary trials are examined for a different mutation rate μ and target number *N_tar_* = 10 (A), 8 (B), and 7 (C), appearance of long and short germ modes are counted respectively at 2000th evolutionary generation. The frequencies are plotted against μ. The ratio of the frequencies, i.e., the frequency of long germ modes divided by that of the short germ modes, is shown in Fig. 4C.
(0.11 MB PDF)

Figure S12 Developmental Robustness of Evolved Networks. Robustness of gene expression pattern against perturbations to the initial conditions (left figure in A–C) was studied. The expression patterns of gene #1 at several time points are shown. Black lines indicate the unperturbed development shown in Figure 1D–F. The variations of the patterns arising from different initial conditions are filtered out during development in all three modes. Simultaneous stripe formation (A and anterior region of B) shows a relatively smaller variation than the sequential one (posterior region of B and C).
(0.17 MB PDF)

Figure S13 Network Modules Included in Core Networks. Decomposition of networks into modules are demonstrated. The networks are shown in Figure 2A–C. (A) There are five FFLs connected in parallel and a FFL connected in series. (B) There are a FFL connected in series, a negative FBL and a positive FBL. (C) There are four FFLs connected in parallel, a FFL connected in series, a negative FBL and positive FBL. By systematically decomposing the other evolved networks into the modules, statistics in Figures 2D and S7A–B were measured.
(0.10 MB PDF)

Figure S14 Evolutionary Parameter Tuning of FFLs Leads to More Regular Stripes. (A)–(C) Evolution of Coefficient of Variation (CV) of peak-to-peak distance in the striped pattern (See Methods S1). Average of the CV among the selected embryos is plotted against generation steps in A. Five representative trials each of which adopts a different network connection yielding long germ mode development. Striped pattern after each evolution is shown in B. In C, the evolution is applied independently to the all networks within long (green) and short (pink) germ modes. Dotted and solid lines indicate frequency of CV before and after the evolution, respectively. Shaded region in A and C indicates CV calculated from striped pattern of *D. melanogaster* in D. The CVs in long germ modes decrease to the same level as those in short germ modes as well as in *D. melanogaster*. For 60% of the long germ networks, the CV after the evolution is not over that in *D. melanogaster*. (D) The pair-rule gene expression patterns of *D. melanogaster* (Methods S1). Seven stripes with larger expression level were adopted to calculate CV and the other smaller peaks were not adopted.
(0.17 MB PDF)

## References

[1] Arthur W (1998). The Origin of Animal Body Plans: A Study in Evolutionary Developmental Biology Cambridge Univ.

[2] Hall BK (1998). Evolutionary Developmental Biology Chapman & Hall.

[3] Kirschner M, Gerhart J (1998). Evolvability.. Proc Natl Acad Sci U S A.

[4] Carroll SB, Grenier JK, Weatherbee SD (2001). From DNA to Diversity: Molecular Genetics and the Evolution of Animal Design: Blackwell Science Inc.

[5] Müller GB, Newman SA (2003). Origination of Organismal Form: Beyond the Gene in Developmental and Evolutionary Biology Bradford Books.

[6] Davidson EH, Erwin DH (2006). Gene regulatory networks and the evolution of animal body plans.. Science.

[7] Sander K (1976). Specification of the basic body pattern in insect embryogenesis.. Adv Insect Physiol.

[8] Davis GK, Patel NH (2002). Short, long, and beyond: molecular and embryological approaches to insect segmentation.. Annu Rev Entomol.

[9] Ingham PW (1988). The molecular genetics of embryonic pattern formation in *Drosophila*.. Nature.

[10] Sommer RJ, Tautz D (1993). Involvement of an orthologue of the *Drosophila* pair-rule gene *hairy* in segment formation of the short germ-band embryo of *Tribolium* (Coleoptera).. Nature.

[11] Stollewerk A, Schoppmeier M, Damen WG (2003). Involvement of *Notch* and *Delta* genes in spider segmentation.. Nature.

[12] Mito T, Okamoto H, Shinahara W, Shinmyo Y, Miyawaki K (2006). *Krüppel* acts as a gap gene regulating expression of *hunchback* and *even-skipped* in the intermediate germ cricket *Gryllus bimaculatus*.. Dev Biol.

[13] Liu PZ, Kaufman TC (2004). *Krüppel* is a gap gene in the intermediate germband insect *Oncopeltus fasciatus* and is required for development of both blastoderm and germband-derived segments.. Development.

[14] Peel AD, Chipman AD, Akam M (2005). Arthropod segmentation: beyond the Drosophila paradigm.. Nat Rev Genet.

[15] Liu PZ, Kaufman TC (2005). Short and long germ segmentation: unanswered questions in the evolution of a developmental mode.. Evol Dev.

[16] Damen WG (2007). Evolutionary conservation and divergence of the segmentation process in arthropods.. Dev Dyn.

[17] Wittkopp PJ (2007). Variable gene expression in eukaryotes: a network perspective.. J Exp Biol.

[18] Kauffman SA (1993). The Origins of Order: Self-Organization and Selection in Evolution: Oxford University Press.

[19] Hogeweg P (2000). Evolving mechanisms of morphogenesis: on the interplay between differential adhesion and cell differentiation.. J Theor Biol.

[20] Bornholdt S, Sneppen K (2000). Robustness as an evolutionary principle.. Proc Biol Sci.

[21] Salazar-Ciudad I, Newman SA, Solé RV (2001). Phenotypic and dynamical transitions in model genetic networks. I. Emergence of patterns and genotype-phenotype relationships.. Evol Dev.

[22] Siegal ML, Bergman A (2002). Waddington's canalization revisited: developmental stability and evolution.. Proc Natl Acad Sci U S A.

[23] Kaneko K (2007). Evolution of robustness to noise and mutation in gene expression dynamics.. PLoS ONE.

[24] Kraft R, Jackle H (1994). *Drosophila* mode of metamerization in the embryogenesis of the lepidopteran insect *Manduca sexta*.. Proc Natl Acad Sci U S A.

[25] Goltsev Y, Hsiong W, Lanzaro G, Levine M (2004). Different combinations of gap repressors for common stripes in *Anopheles* and *Drosophila* embryos.. Dev Biol.

[26] Davis GK, Jaramillo CA, Patel NH (2001). Pax group III genes and the evolution of insect pair-rule patterning.. Development.

[27] Dearden PK, Akam M (2001). Early embryo patterning in the grasshopper, *Schistocerca gregaria*: *wingless*, *decapentaplegic* and *caudal* expression.. Development.

[28] Copf T, Rabet N, Celniker SE, Averof M (2003). Posterior patterning genes and the identification of a unique body region in the brine shrimp *Artemia franciscana*.. Development.

[29] Chipman AD, Arthur W, Akam M (2004). A double segment periodicity underlies segment generation in centipede development.. Curr Biol.

[30] Choe CP, Miller SC, Brown SJ (2006). A pair-rule gene circuit defines segments sequentially in the short-germ insect *Tribolium castaneum*.. Proc Natl Acad Sci U S A.

[31] Liu PZ, Kaufman TC (2005). *even-skipped* is not a pair-rule gene but has segmental and gap-like functions in *Oncopeltus fasciatus*, an intermediate germband insect.. Development.

[32] Shen-Orr SS, Milo R, Mangan S, Alon U (2002). Network motifs in the transcriptional regulation network of *Escherichia coli*.. Nat Genet.

[33] Lee TI, Rinaldi NJ, Robert F, Odom DT, Bar-Joseph Z (2002). Transcriptional regulatory networks in *Saccharomyces cerevisiae*.. Science.

[34] Wall ME, Hlavacek WS, Savageau MA (2004). Design of gene circuits: lessons from bacteria.. Nat Rev Genet.

[35] Alon U (2006). An Introduction to Systems Biology: Design Principles of Biological Circuits: Chapman & Hall.

[36] Cordero OX, Hogeweg P (2006). Feed-forward loop circuits as a side effect of genome evolution.. Mol Biol Evol.

[37] Chickarmane V, Troein C, Nuber UA, Sauro HM, Peterson C (2006). Transcriptional dynamics of the embryonic stem cell switch.. PLoS Comput Biol.

[38] Baumgardt M, Miguel-Aliaga I, Karlsson D, Ekman H, Thor S (2007). Specification of neuronal identities by feedforward combinatorial coding.. PLoS Biol.

[39] Tsang J, Zhu J, van Oudenaarden A (2007). MicroRNA-mediated feedback and feedforward loops are recurrent network motifs in mammals.. Mol Cell.

[40] Shalgi R, Lieber D, Oren M, Pilpel Y (2007). Global and Local Architecture of the Mammalian microRNA-Transcription Factor Regulatory Network.. PLoS Comput Biol.

[41] Lau KY, Ganguli S, Tang C (2007). Function constrains network architecture and dynamics: a case study on the yeast cell cycle Boolean network.. Phys Rev E Stat Nonlin Soft Matter Phys.

[42] Ishihara S, Fujimoto K, Shibata T (2005). Cross talking of network motifs in gene regulation that generates temporal pulses and spatial stripes.. Genes Cells.

[43] Isalan M, Lemerle C, Serrano L (2005). Engineering gene networks to emulate *Drosophila* embryonic pattern formation.. PLoS Biol.

[44] Basu S, Gerchman Y, Collins CH, Arnold FH, Weiss R (2005). A synthetic multicellular system for programmed pattern formation.. Nature.

[45] Pourquié O (2003). The segmentation clock: converting embryonic time into spatial pattern.. Science.

[46] Horikawa K, Ishimatsu K, Yoshimoto E, Kondo S, Takeda H (2006). Noise-resistant and synchronized oscillation of the segmentation clock.. Nature.

[47] Masamizu Y, Ohtsuka T, Takashima Y, Nagahara H, Takenaka Y (2006). Real-time imaging of the somite segmentation clock: revelation of unstable oscillators in the individual presomitic mesoderm cells.. Proc Natl Acad Sci U S A.

[48] Meinhardt H (1982). Models of Biological Pattern Formation Academic Press.

[49] Salazar-Ciudad I, Solé RV, Newman SA (2001). Phenotypic and dynamical transitions in model genetic networks. II. Application to the evolution of segmentation mechanisms.. Evol Dev.

[50] Nüsslein-Volhard C, Wieschaus E (1980). Mutations affecting segment number and polarity in *Drosophila*.. Nature.

[51] Ishihara S, Shibata T (2008). Mutual interaction in network motifs robustly sharpens gene expression in developmental processes.. J Theor Biol.

[52] von Dassow G, Meir E, Munro EM, Odell GM (2000). The segment polarity network is a robust developmental module.. Nature.

[53] Jaeger J, Surkova S, Blagov M, Janssens H, Kosman D (2004). Dynamic control of positional information in the early *Drosophila* embryo.. Nature.

[54] Mito T, Kobayashi C, Sarashina I, Zhang H, Shinahara W (2007). *even-skipped* has gap-like, pair-rule-like, and segmental functions in the cricket *Gryllus bimaculatus*, a basal, intermediate germ insect (Orthoptera).. Dev Biol.

[55] Jaynes JB, Fujioka M (2004). Drawing lines in the sand: *even skipped et al.* and parasegment boundaries.. Dev Biol.

[56] Nüsslein-Volhard C, Kluding H, Jurgens G (1985). Genes affecting the segmental subdivision of the *Drosophila* embryo.. Cold Spring Harb Symp Quant Biol.

[57] Brown SJ, Denell RE (1996). Segmentation and dorsal-ventral patterning in *Tribolium*.. Sem Cell Dev Biol.

[58] Milo R, Itzkovitz S, Kashtan N, Levitt R, Shen-Orr S (2004). Superfamilies of evolved and designed networks.. Science.

[59] Patel NH, Ball EE, Goodman CS (1992). Changing role of *even-skipped* during the evolution of insect pattern formation.. Nature.

[60] Barkai N, Leibler S (1997). Robustness in simple biochemical networks.. Nature.

[61] de Visser JA, Hermisson J, Wagner GP, Ancel Meyers L, Bagheri-Chaichian H (2003). Perspective: Evolution and detection of genetic robustness.. Evolution Int J Org Evolution.

[62] Ma W, Lai L, Ouyang Q, Tang C (2006). Robustness and modular design of the *Drosophila* segment polarity network.. Mol Syst Biol.

[63] Ciliberti S, Martin OC, Wagner A (2007). Robustness can evolve gradually in complex regulatory gene networks with varying topology.. PLoS Comput Biol.

[64] Schroder R (2003). The genes *orthodenticle* and *hunchback* substitute for *bicoid* in the beetle *Tribolium*.. Nature.

[65] Bucher G, Klingler M (2004). Divergent segmentation mechanism in the short germ insect *Tribolium* revealed by *giant* expression and function.. Development.

[66] Cerny AC, Bucher G, Schroder R, Klingler M (2005). Breakdown of abdominal patterning in the *Tribolium Krüppel* mutant *jaws*.. Development.

[67] Savard J, Marques-Souza H, Aranda M, Tautz D (2006). A segmentation gene in *tribolium* produces a polycistronic mRNA that codes for multiple conserved peptides.. Cell.

[68] Monod J (1972). Chance and Necessity: Collins.

[69] Kaneko K (2006). Life: An Introduction to Complex Systems Biology.

[70] Francois P, Hakim V, Siggia ED (2007). Deriving structure from evolution: metazoan segmentation.. Mol Syst Biol.

[71] Cooke J, Zeeman EC (1976). A clock and wavefront model for control of the number of repeated structures during animal morphogenesis.. J Theor Biol.

[72] Driever W, Nusslein-Volhard C (1988). A gradient of *bicoid* protein in *Drosophila* embryos.. Cell.

[73] Gregor T, Bialek W, de Ruyter van Steveninck RR, Tank DW, Wieschaus EF (2005). Diffusion and scaling during early embryonic pattern formation.. Proc Natl Acad Sci U S A.

[74] Damen WG, Weller M, Tautz D (2000). Expression patterns of *hairy*, *even-skipped*, and *runt* in the spider *Cupiennius salei* imply that these genes were segmentation genes in a basal arthropod.. Proc Natl Acad Sci U S A.

[75] Davis GK, D'Alessio JA, Patel NH (2005). Pax3/7 genes reveal conservation and divergence in the arthropod segmentation hierarchy.. Dev Biol.

