# Supplementary material for: Network Evolution of Body Plans
Source: PLoS One. 2008 Jul 23;3(7):e2772. doi: 10.1371/journal.pone.0002772 (PMC2464711; doi:10.1371/journal.pone.0002772)
Supplement: Methods S1 — (0.13 MB PDF) [file pone.0002772.s001.pdf]

“Network Evolution of Body Plans”
by Koichi Fujimoto, Shuji Ishihara, and Kunihiko Kaneko

Supporting Methods S1.

Implementation of experiments:

All numerical calculations were implemented in C. Equation 1 in Methods was integrated using the Euler method. We verified that the obtained results are little influenced by the choice of step size in time and space. The typical CPU time for calculating 1000 generations of a single evolutionary pathway is approximately 2 hours (Athlon 2.8GHz CPU; AMD). Network topologies (Figs. 2A-C, S4 and S5) were drawn by Pajek (*http://vlado.fmf.uni-lj.si/pub/networks/pajek/*).

Network size:

In the present paper, the number of genes and regulatory connections are fixed to 32 and 64, respectively. We have confirmed that the present results hold when the numbers of genes and connections are changed to 16 and 32, and 64 and 128. According to literature-based databases, the connection density, i.e., the number of the connection divided by the number of the genes in transcription [1], is about 2 for early development of *D. melanogaster* [2-4], and 1~1.5 for those of yeast *S. Cerevisiae* [3,5] and bacteria *E. Coli* [6]. We have also confirmed that the behavior of the system and the results presented here are preserved for a connection density between 1.5 and 4. Also, the results are not affected when the number of nodes and connections are allowed to change by mutation during evolution.

Regulation by multiple genes:

Consider that gene #i is regulated by a single gene #j, then mutation adds a second regulator #k for gene #i. For such a mutation, we randomly adopt cooperative or competitive regulation [7,8] between #j and #k. These regulations are represented by

$$\frac{\partial P_i}{\partial t} = \begin{cases} f(P_j; K_{j\to i}) \cdot f(P_k; K_{k\to i}) - \gamma P_i + D_i \dfrac{\partial^2 P_i}{\partial x^2} & \text{(cooperative)} \\ \text{or} & \\ f(P_j; K_{j\to i}) + f(P_k; K_{k\to i}) - \gamma P_i + D_i \dfrac{\partial^2 P_i}{\partial x^2} & \text{(competitive)} \end{cases} \qquad \text{(S1)}$$

When a gene #i is regulated by more that three genes #j, #k, and #l, the adopted regulation is a combination of cooperative and competitive ones, e.g.

$$\frac{\partial P_i}{\partial t} = f(P_j; K_{j\to i}) \cdot f(P_k; K_{k\to i}) + f(P_l; K_{l\to i}) - \gamma P_i + D_i \frac{\partial^2 P_i}{\partial x^2} \tag{S2}$$

Maternal factor:

We assigned gene #0 as a maternal factor under no influence from other genes. It is translated at the anterior end of an embryo, and the product is allowed to diffuse and degrade. Thus dynamics of the spatial pattern formation obeys following equation.

$$\frac{\partial P_0}{\partial t} = -\gamma P_0 + D_0 \frac{\partial^2 P_0}{\partial x^2}$$

with the boundary condition $P_0(x=0)=A$. It establishes exponential gradient in space [9,10].

$$P_0(x) = A \exp\left(-\sqrt{\frac{\gamma}{D_0}}x\right) \tag{S3}$$

Mutation:

As described in Method, mutations are introduced in network elements: (A) a connection matrix $c_{j\to i}$, (B) a threshold $K_{j\to i}$ and (C) a diffusion constant D_i. (A) A connection is rewired by fixing one of the two nodes and at the same time avoiding an overlap with existing connections. During rewiring, the connection may flip from a positive to a negative regulation or vice versa at random. (B)To introduce mutation to a threshold in a connection from gene #j to #i, the value of $K_{j\to i}$ is randomized in a range $0.01 \leq K_{j\to i} \leq 10$. (C) The value of D_i is randomized in a range $0.1 \leq D_i \leq 1$. Probability of each mutation in a network is given by the mutation rate μ. Since the total number of mutant networks per generation is given by $N_S N_m$, the expected number of mutant networks is $\mu N_S N_m$ per generation.

Evolution of regular striped pattern by parameter tuning:

The peak-to-peak distance of stripes in long germ mode was rather heterogeneous in the present simulation (e.g., Figs. 1D and S5A). To examine if the heterogeneity is relaxed by tuning the parameters in the expression dynamics, we carried out numerical

evolution by further imposing the selection pressure to homogenize the peak-to-peak distance of the stripe of the expression level of #1. To be specific we introduced Coefficient of Variation (CV) in peak-to-peak distance, given by

$$\begin{cases} CV \equiv \dfrac{\sigma_\Delta}{\bar{\Delta}} \\ \bar{\Delta} \equiv \sum_i \Delta_i, \ \sigma_\Delta \equiv \sqrt{\sum_i \left(\Delta_i - \bar{\Delta}\right)^2} \end{cases}$$

where Δ_i indicates distance between *i*-th and *i+1*-th peaks (see Figure S14A and B for evolution and definition of CV). The smaller CV has a higher fitness. As an initial condition of the evolution, we adopted a network within long germ mode (e.g, Figs. 2A and S3A). Mutations are introduced only to parameters $K_{j \to i}$ with mutation rate μ and the network topology is fixed, in contrast to other evolutionary simulations in the present paper. Total number of mutants per generation is given by $N_S N_m$ and top N_S mutants having lower values of the CV are selected. The evolved CV values for long germ mode are also compared with those of short germ mode (Fig. S14C) as well as those in pair-rule gene expression of *D. melanogaster* (Fig. S14D). Ensembles of initial networks used to calculate frequency of CV in long and short germ modes (dotted lines in Fig. S14C) are identical with those used in Figure 2D. The quantitative expression patterns of *D. melanogaste*r were downloaded from FlyEx (*http://flyex.ams.sunysb.edu/FlyEx/*) [11], where data were integrated with 10% strip in dorso-ventral direction without background [12]. The number of data points in antero-posterior direction is 100, same as in the present model. The data are based on class 8 for the 14th cleavage cycle during embryogenesis.

1. Kauffman SA (1993) The Origins of Order: Self-Organization and Selection in Evolution: Oxford University Press.
2. Serov VN, Spirov AV, Samsonova MG (1998) Graphical interface to the genetic network database GeNet. Bioinformatics 14: 546-547.
3. Rosenfeld N, Alon U (2003) Response delays and the structure of transcription networks. J Mol Biol 329: 645-654.
4. Ishihara S, Fujimoto K, Shibata T (2005) Cross talking of network motifs in gene regulation that generates temporal pulses and spatial stripes. Genes Cells 10: 1025-1038.
5. Costanzo MC, Crawford ME, Hirschman JE, Kranz JE, Olsen P, et al. (2001) YPD, PombePD and WormPD: model organism volumes of the BioKnowledge library, an integrated resource for protein information. Nucleic Acids Res 29: 75-79.
6. Shen-Orr SS, Milo R, Mangan S, Alon U (2002) Network motifs in the transcriptional regulation network of *Escherichia coli*. Nat Genet 31: 64-68.
7. Setty Y, Mayo AE, Surette MG, Alon U (2003) Detailed map of a *cis*-regulatory input function. Proc Natl Acad Sci U S A 100: 7702-7707.
8. Buchler NE, Gerland U, Hwa T (2003) On schemes of combinatorial transcription logic. Proc Natl Acad Sci U S A 100: 5136-5141.
9. Houchmandzadeh B, Wieschaus E, Leibler S (2002) Establishment of developmental precision and proportions in the early *Drosophila* embryo. Nature 415: 798-802.
10. Gregor T, Bialek W, de Ruyter van Steveninck RR, Tank DW, Wieschaus EF (2005) Diffusion and scaling during early embryonic pattern formation. Proc Natl Acad Sci U S A 102: 18403-18407.
11. Poustelnikova E, Pisarev A, Blagov M, Samsonova M, Reinitz J (2004) A database for management of gene expression data in situ. Bioinformatics 20: 2212-2221.
12. Myasnikova E, Samsonova A, Kozlov K, Samsonova M, Reinitz J (2001) Registration of the expression patterns of Drosophila segmentation genes by two independent methods. Bioinformatics 17: 3-12.
