# Supplementary material for: Network Evolution of Body Plans
Source: PLoS One. 2008 Jul 23;3(7):e2772. doi: 10.1371/journal.pone.0002772 (PMC2464711; doi:10.1371/journal.pone.0002772)
Supplement: Result S1 — Theoretical Analyses of Gene Regulation in a Long Germ Network. (0.06 MB PDF) [file pone.0002772.s002.pdf]

“Network Evolution of Body Plans”
by Koichi Fujimoto, Shuji Ishihara, and Kunihiko Kaneko

<u>Supporting Result S1.</u>
Theoretical Analyses of Gene Regulation in a Long Germ Network.

For a given regulatory networks without any FBL, “Gene Regulation Function[1-3]” (GRF) is useful to understand how the network generates stripe gene expressions in space. GRF is the function that plots the expression level of a regulated gene against its regulator. Here the regulator can be anywhere in the upstream of the regulation (i.e., indirect regulations are allowed). In this section, we give an example of the analysis by GRF on the network shown in Fig. 2A (see also Fig. S7).

Let us begin with the simplest case – a direct interaction between gene #5 and gene #0 in Fig. 2A. The GRF is given by

$$P_5 = f_+(P_0; K_{0\to5}) = \frac{P_0^{\alpha}}{P_0^{\alpha} + K_{0\to5}{}^{\alpha}}$$

This function can be analytically derived from equation 1 at the steady state, i.e. when $\frac{dP_5}{dt} = 0$ and $\frac{\partial^2 P_5}{\partial x^2} = 0$.
We shall hereafter refer to the positive and negative gene regulation functions in equation 1 as f_+ and f_-, respectively.

If there are more than two regulatory connections to a gene, there could be multiple regulatory pathways from an upstream gene to the gene via FFLs. The “decomposed GRF” introduced below elucidates the role of multiple regulations. Let us consider a FFL (marked by + in Fig. 2A; also Fig. S7A) from #0 to # 30 as an example. First, following equation S1 in Methods S1, combinatorial regulation for gene #30 is decomposed into elementary regulation denoted by $P_{30}=f_+(P_{26};K_{26\to30})$ and $P_{30}=f_-(P_5;K_{5\to30})$. Next, the expression level of the gene #0 that regulates #26 and #5 is substituted into the decomposed GRFs. They are expressed by P_0 as

$$P_{30} = f_+\left(f_+(P_0; K_{0\to26}); K_{26\to30}\right)$$

(red dotted line in Fig. S7A) and

$$P_{30} = f_{-}\left(f_{+}(P_0; K_{0\to 5}); K_{5\to 30} \right)$$

(blue dashed line in Fig. S7A), respectively. Finally, when combinatorial regulation (eq. S1) of gene #30 by #5 and #26 is taken into account, the steady state yields

$$P_{30} = f_{-}(P_5; K_{5\to 30}) \cdot f_{+}(P_{26}; K_{26\to 30})$$

Furthermore the “integrated GRF” of P_{30} is expressed as a function of P_0 by

$$P_{30} = f_{-}\left(f_{+}(P_0; K_{0\to 5}); K_{5\to 30} \right) \cdot f_{+}\left(f_{+}(P_0; K_{0\to 26}); K_{26\to 30} \right) \qquad \text{(S4)}$$

It follows that this function shows a bell-shaped concave curve (a solid line in Fig. S7A). For a spatial pattern, a GRF can also be numerically obtained from expression level of genes measured at each position [2,3] (a dotted line in Fig. S8). A GRF, that is measured from a single stripe pattern of gene #30 and a gradient pattern of gene #0 (Fig. S2A), agrees well with the integrated GRF analytically derived above (Fig. S8).

When the procedure is recursively applied to include the downstream genes, the final decomposed and integrated GRFs become functions of P_0. Regulation of gene #6 by gene #0 is assembled by three pathways (marked in violet, red, and light blue lines in Figure S7B). Since gene #30 negatively regulates #6, the decomposed GRF for a pathway via the above FFL shows a convex bell-shaped curve (a violet line in Figure S7B) denoted by

$$P_6 = f_{-}\left(f_{-}\left(f_{+}(P_0; K_{0\to 5}); K_{5\to 30} \right) \cdot f_{+}\left(f_{+}(P_0; K_{0\to 26}); K_{26\to 30} \right); K_{30\to 6} \right)$$

The other decomposed GRFs represent activation (a red line in Figure S7B),

$$P_6 = f_{+}\left(f_{+}(P_0; K_{0\to 26}); K_{26\to 6} \right)$$

and repression (a light blue line in Figure S7B),

$$P_6 = f_-\left(f_+(P_0; K_{0\to10}); K_{10\to6}\right)$$

Combinatorial regulation (eq. S2 in Methods S1) of gene #6 by genes #30, #26, and #10 is given by

$$P_6 = f_+(P_{26}; K_{26\to6}) \cdot f_-(P_{30}; K_{30\to6}) + f_-(P_{10}; K_{10\to6})$$

The integrated GRF of P_6 as a function of P_0 is therefore

$$\begin{aligned} P_6 &= f_+\left(f_+(P_0; K_{0\to26}); K_{26\to6}\right) \\ &\cdot f_-\left(f_-\left(f_+(P_0; K_{0\to5}); K_{5\to30}\right) \cdot f_+\left(f_+(P_0; K_{0\to26}); K_{26\to30}\right); K_{30\to6}\right) \\ &+ f_-\left(f_+(P_0; K_{0\to10}); K_{10\to6}\right) \end{aligned} \quad \text{(S5)}$$

This has two local minima (solid line in Fig. S7B) resulting in the spatial pattern with two local minima (gene #6 in Fig. S2A) because $P_0(x)$ is monotonically decreasing function (eq. S3 in Methods S1 and Fig. S2A).

In Figure S7C, there are three regulatory connections to gene #14 from genes #17, #6, and #2. A decomposed GRF as a function of P_0 is calculated by successively substituting equation S4 integrated GRFs of P_{30} and to $P_{17}=f_-(P_{30};K_{30\to17})$, then $P_{14}=f_+(P_{17};K_{17\to14})$ (an orange line), and equation S5 to $P_{14}=f_-(P_6;K_{6\to14})$ (a blue line) and $P_4=f_-(P_6;K_{6\to4})$, $P_2=f_+(P_4;K_{4\to2})$, then $P_{14}=f_-(P_2;K_{2\to14})$ (a red line). The combinatorial regulation of #14 is given by

$$P_{14} = f_+(P_{17}; K_{17\to14}) \cdot f_-(P_6; K_{6\to14}) + f_-(P_2; K_{2\to14}) \quad \text{(S6)}$$

The integrated GRF of P_{14} plotted against P_0 shows five local minima (solid line) resulting in five stripes in the spatial pattern (Fig. S2A).

In Figure S7D, a FFL from #14 to #1 (marked by * in Fig. 2A) is decomposed into direct regulation and indirect regulation via #27. The corresponding GRF is calculated by recursively substituting equation S6 either to $P_1=f_+(P_{14};K_{14\to1})$ (a red line) or

$P_{27}=f_{-}(P_{14};K_{14\rightarrow 27})$, then $P_{1}=f_{+}(P_{27};K_{27\rightarrow 1})$ (a blue line), respectively. Since the pathways to gene #1 directly from #14 and indirectly from #14 via #27 are competitive (eq. S1) in the present network.

$$P_1 = f_+(P_{14}; K_{14\rightarrow 1}) + f_+(P_{27}; K_{27\rightarrow 1}) \qquad \text{(S6)}$$

The integrated GRF of P_1 plotted against P_0 shows ten local maxima (solid line) resulting in ten stripes in the spatial pattern (Fig. 1A).

1. Rosenfeld N, Young JW, Alon U, Swain PS, Elowitz MB (2005) Gene regulation at the single-cell level. Science 307: 1962-1965.
2. Ishihara S, Fujimoto K, Shibata T (2005) Cross talking of network motifs in gene regulation that generates temporal pulses and spatial stripes. Genes Cells 10: 1025-1038.
3. Gregor T, Tank DW, Wieschaus EF, Bialek W (2007) Probing the Limits to Positional Information. Cell 130: 153-164.
