# Supplementary figures and images for: Network Evolution of Body Plans
Source: PLoS One. 2008 Jul 23;3(7):e2772. doi: 10.1371/journal.pone.0002772 (PMC2464711; doi:10.1371/journal.pone.0002772)

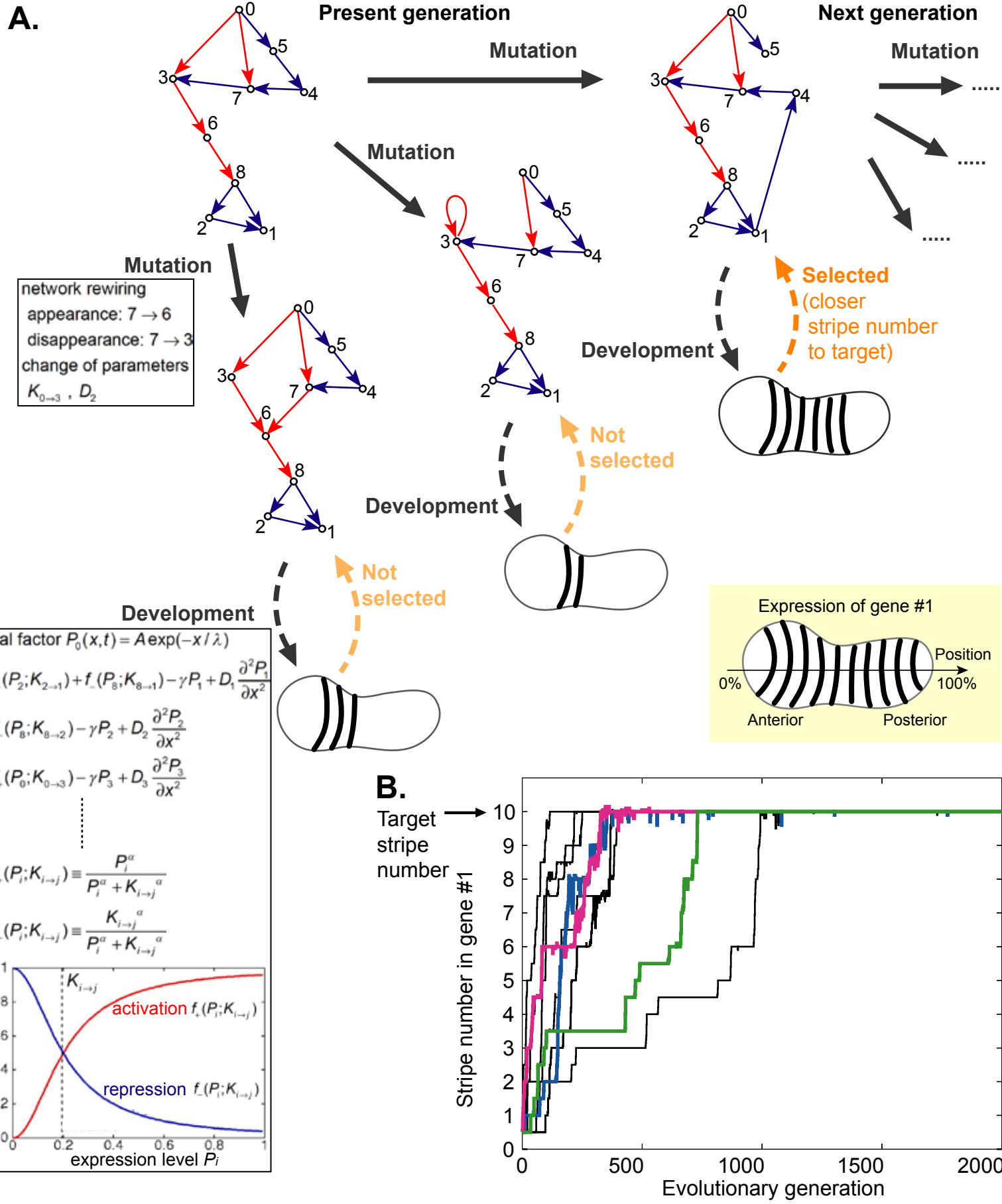

Supplement: Figure S1 — Evolution of the Striped Pattern. (A) Schematic representation of our numerical evolution. A generation is composed of mutation, development, and selection. (B) The average number of stripes for the selected top Ns = 10 embryos, plotted against generation steps. Nine evolutionary trials are shown. The number of the stripes reaches the target Ntar = 10 after several hundred generations, and it is thereafter sustained. The networks selected at 2000th generation after the evolutionary trials plotted in green, pink, and blue exhibit development shown in Fig. 1D–F, respectively. (0.29 MB PDF) [file pone.0002772.s003.pdf]

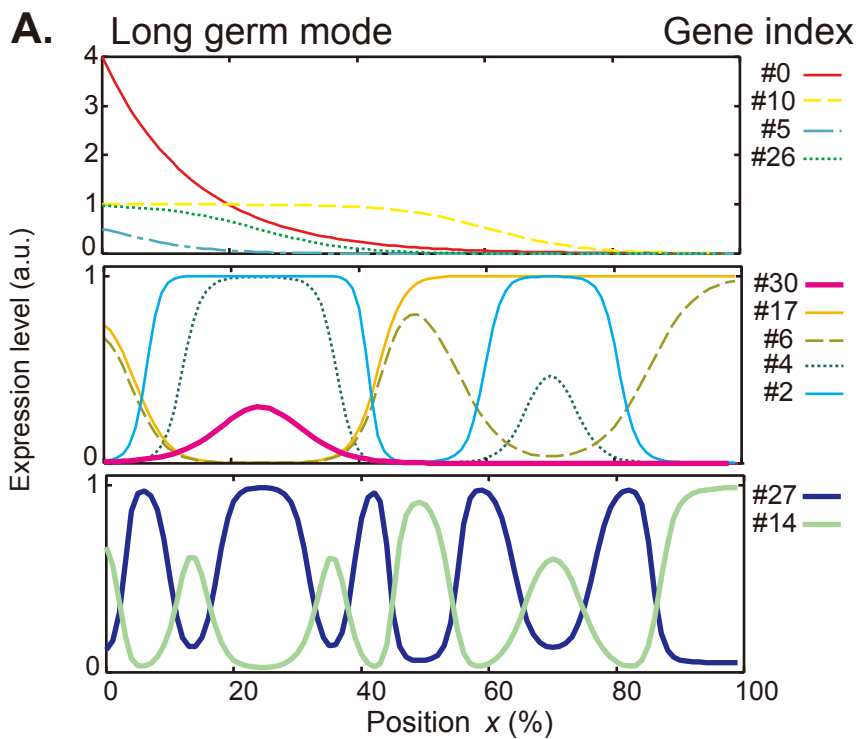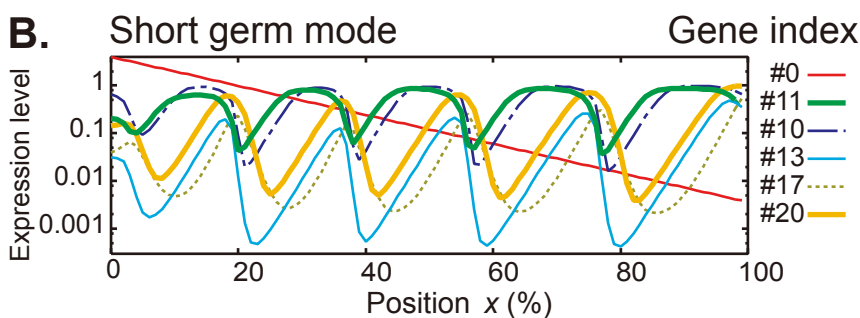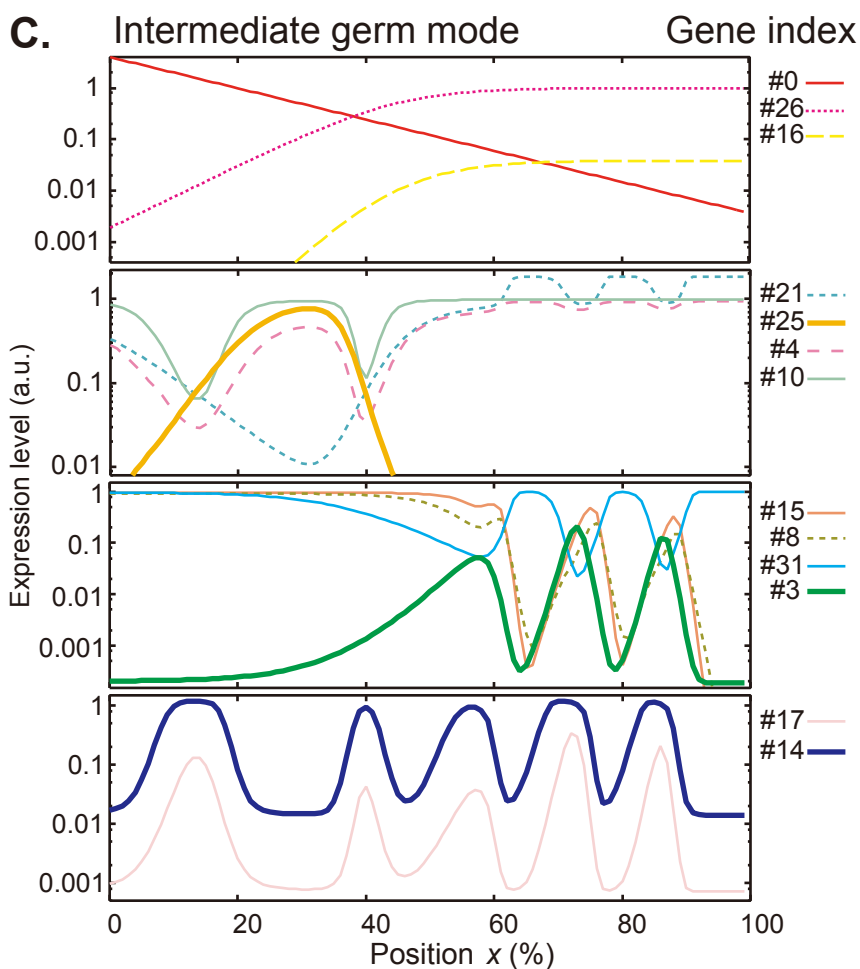

Supplement: Figure S2 — Quantitative Representation of the Final Spatial Profiles of Upstream Gene Expression. (A–C) The final expression levels of genes in the core network (Fig. 2A–C) plotted as a function of position. The Y-axis is in a logarithmic scale for B and C. The color representation for the gene index corresponds to that of the digitized plot of gene expression in Fig. 1G–I, respectively. See the spatio-temporal expression for the genes represented by bold lines in Fig. S3). Gene #0 shown in the uppermost panels in A–C forms a spatial gradient as observed in the maternal genes in arthropods [27], [28], [72], [73]. Gene expression shown in the second panel for A and C is confined to a few domains as in the gap genes [12], [13], [25]. Gene expression patterns shown in the 3rd panel of A and 4th panel of C, and those in B except gene #0 show stripes half as many as those in gene #1, consistent with the pair-rule and segment polarity gene expressions [9], [24], [30], [54]. (0.12 MB PDF) [file pone.0002772.s004.pdf]

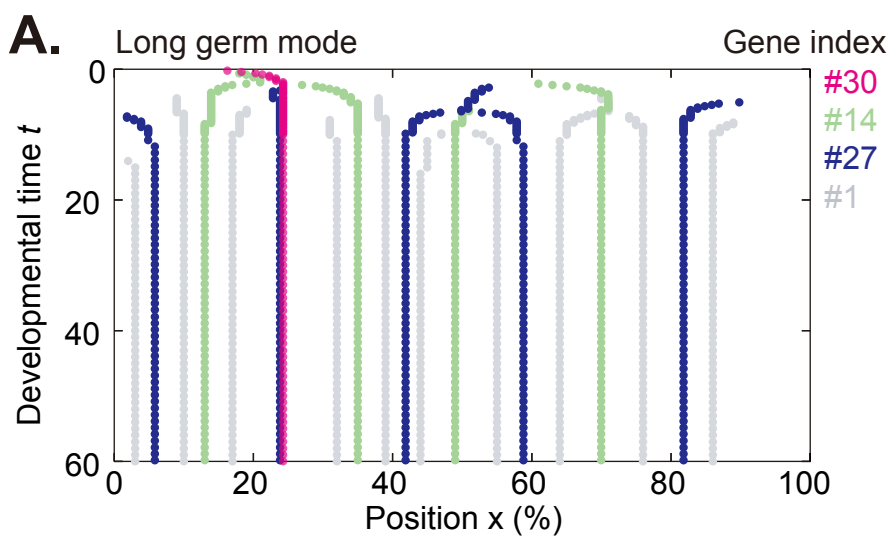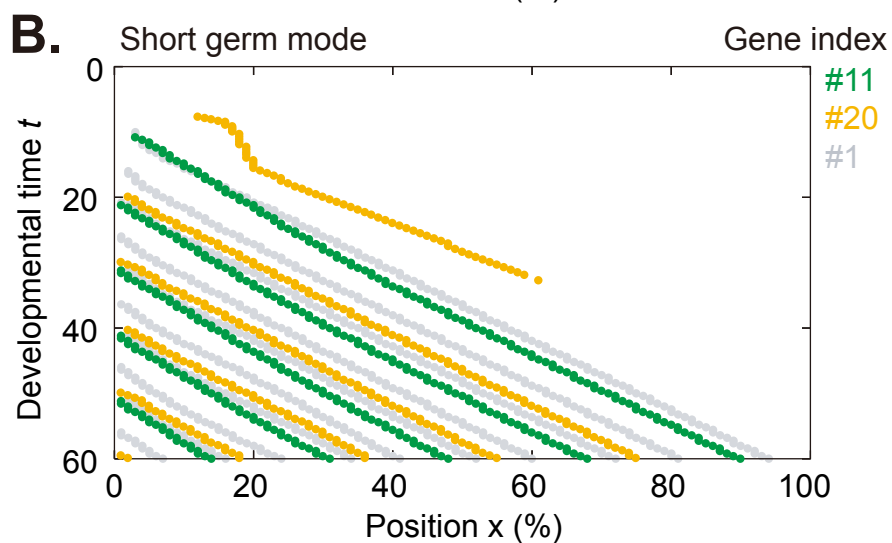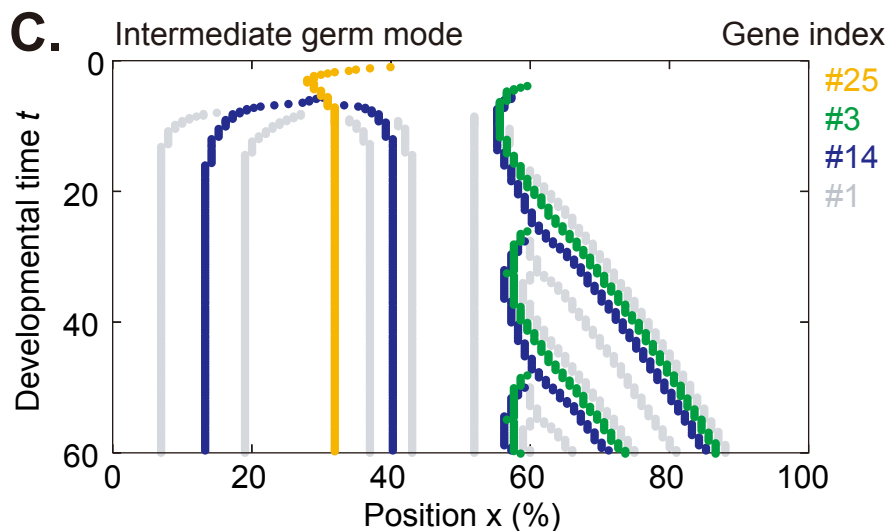

Supplement: Figure S3 — The Developmental Time-course of the Upstream Gene Expression. Local maxima of gene expression are plotted against developmental time, where colors indicate the corresponding genes shown in Figure 1G–I, respectively. (A) Genes begin to be expressed almost simultaneously at 0<t<15 in the order of regulatory pathway (Fig. 2A), i.e., gene #30, #14, #27 and #1. After this transient, the positions of expressed genes are maintained over the subsequent development. (B) Genes organized by a FBL (marked by Δ in Fig. 2B) are expressed sequentially to form stripes in #11 and #20, as in pair-rule genes and Notch/Delta related genes in short germ arthropod [10], [74], [75]. Next, FFL connected in series (* in Fig. 2B) double the stripes into gene #1. (C) First, a FFL (composed of gene #0, #26, #16 and #21 in Fig. 2C) generates a stripe in gene #25. Another FFL (composed of #21, #25 and #4) doubles the stripe in gene #4, resulting simultaneous appearance of two stripes in #14 at x<50. On the other hand, the stripes of gene #3 are formed sequentially by a FBL (marked by Δ in Fig. 2C), resulting sequential appearance of three stripes in #14 at x>50. Serially connected FFL (marked by * in Fig. 2C) doubles the stripes of gene #14 and output to gene #1. The posterior side shows coherent development of gene #3 and #1 as in even-skipped and segment polarity gene expressions in O. fasciatus [31], whereas the anterior side shows coherent pattern of gene #25 and #1 as in Krüppel and segment polarity gene expressions in G. Bimaculatus [12] and O. fasciatus [13]. Knockout experiments confirmed the functionalities of the corresponding network modules (Figs. 3 and S10). (0.38 MB PDF) [file pone.0002772.s005.pdf]

**A.**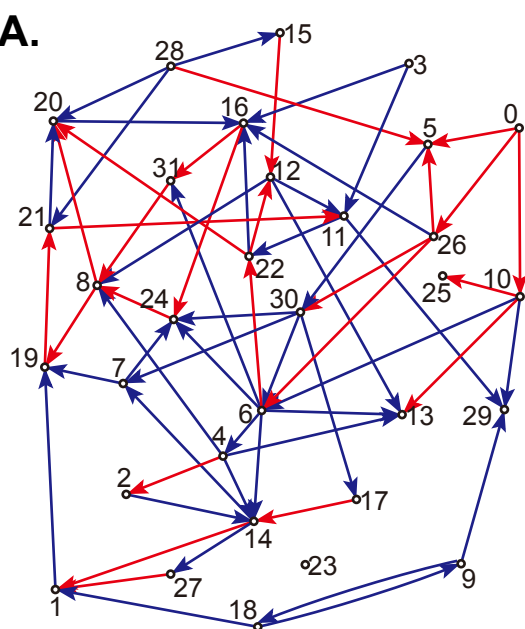**B.**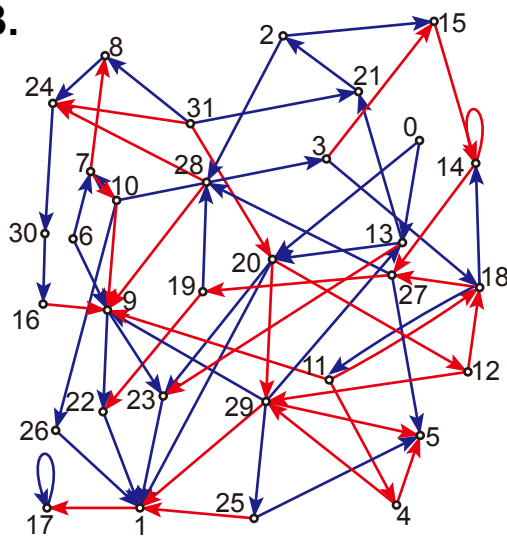**C.**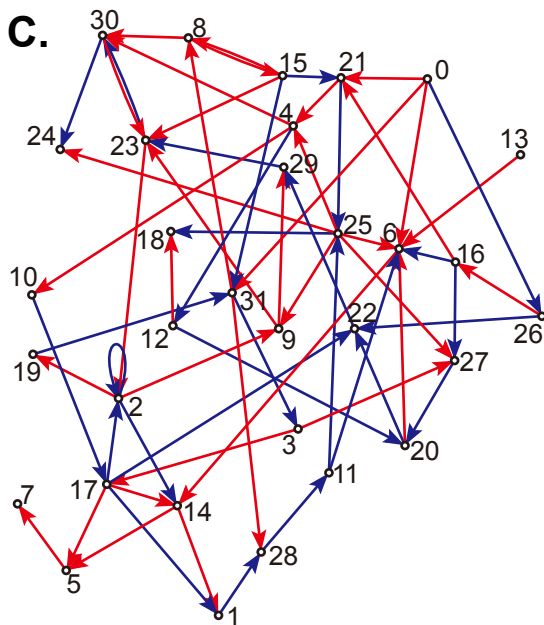**D.**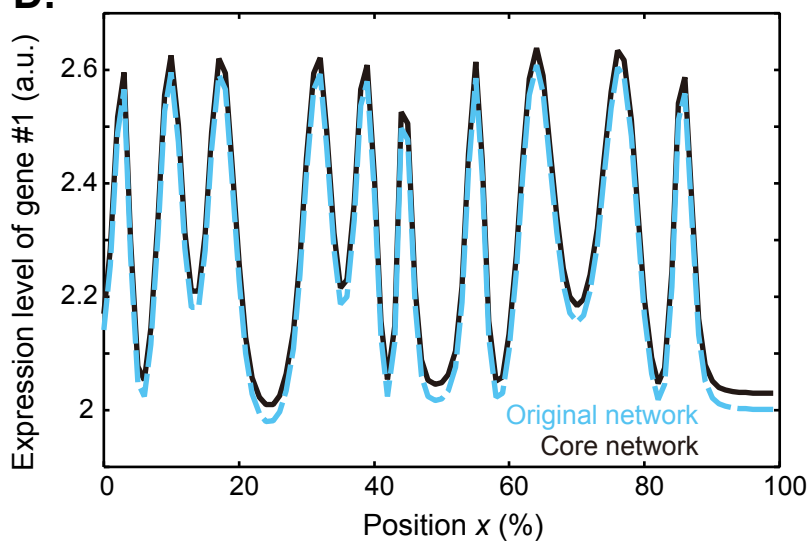**E.**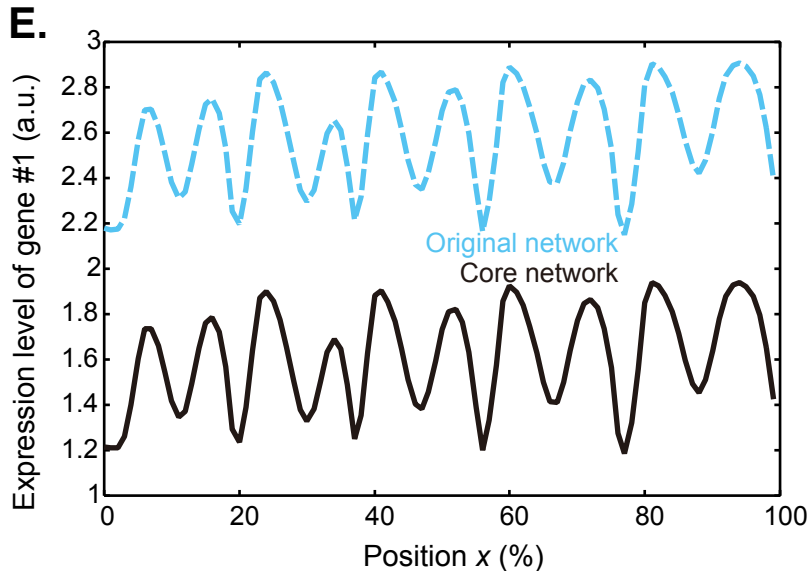**F.**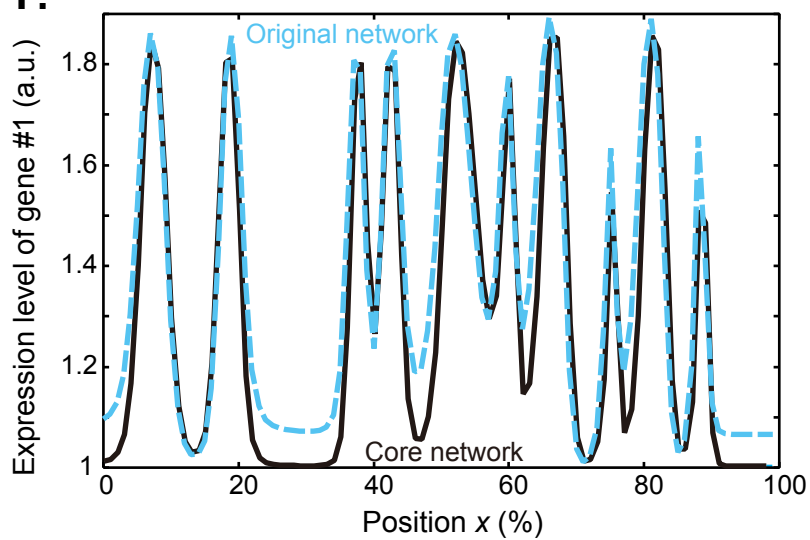

Supplement: Figure S4 — The Core Networks Accurately Reproduce the Developmental Dynamics of the Original Networks. (A–C) The original gene regulatory networks from which the core networks shown in Figure 2A–C were derived. (D–F) Expression pattern of gene #1 generated by the original network (a right blue dashed line) and by the extracted core network (a black solid line, the same as the lower panel in Figure 1D–F, respectively). Not only the number but also the pattern profiles are almost identical in the original and the core networks. The basal level, however, may be shifted as shown in E. (0.14 MB PDF) [file pone.0002772.s006.pdf]

# A. Long germ mode

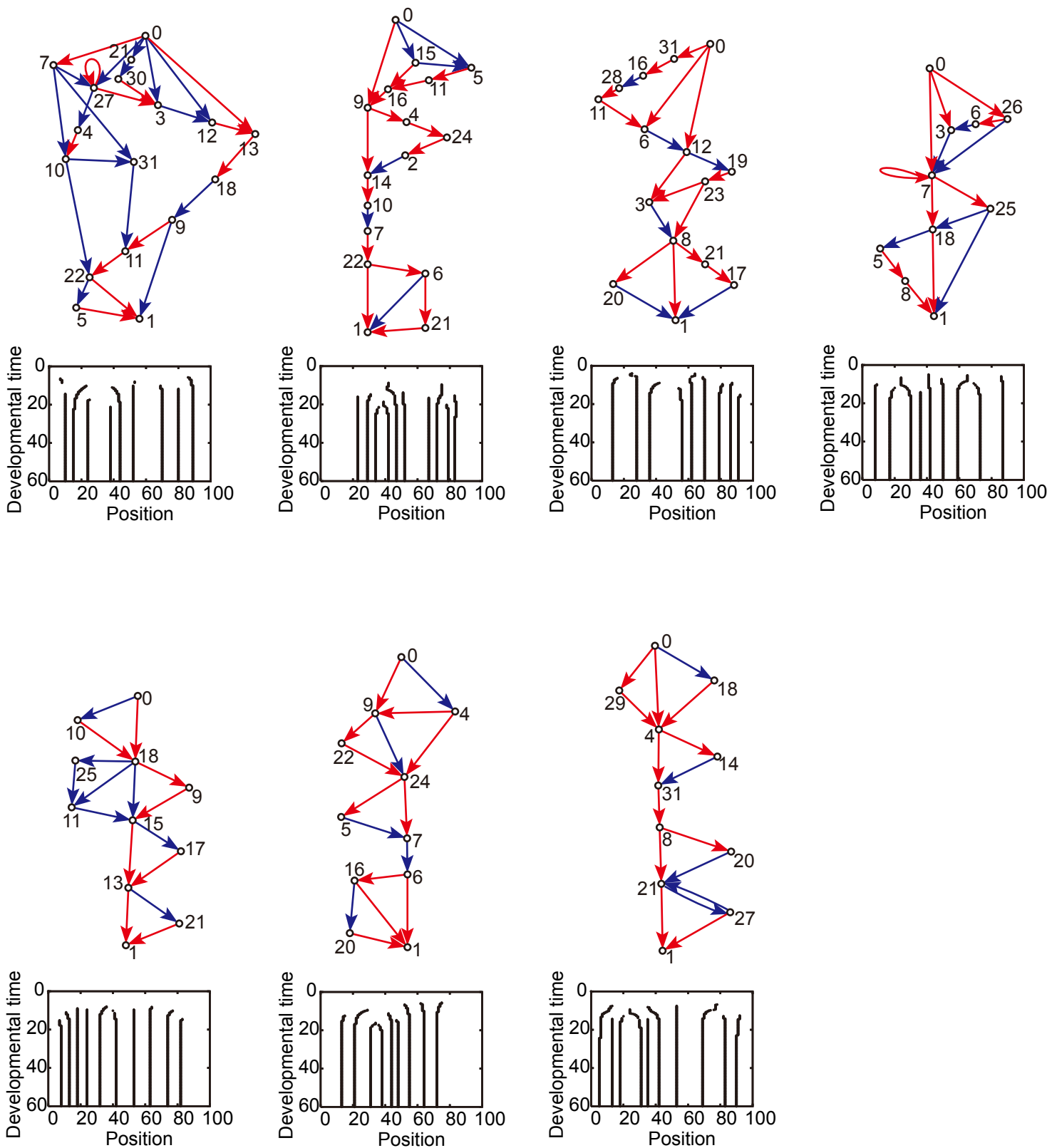

B. Short germ mode

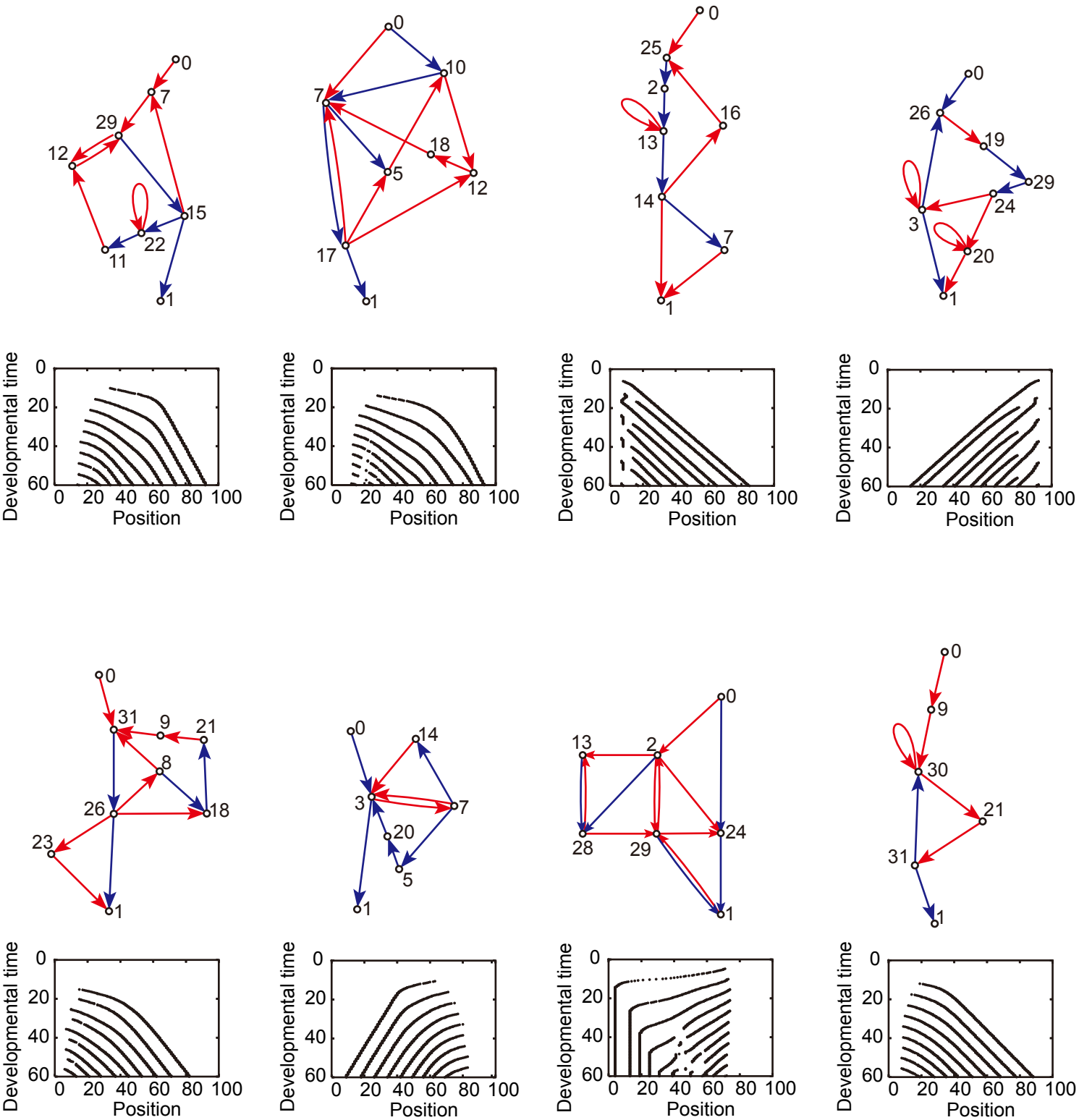

# C. Intermediate germ mode

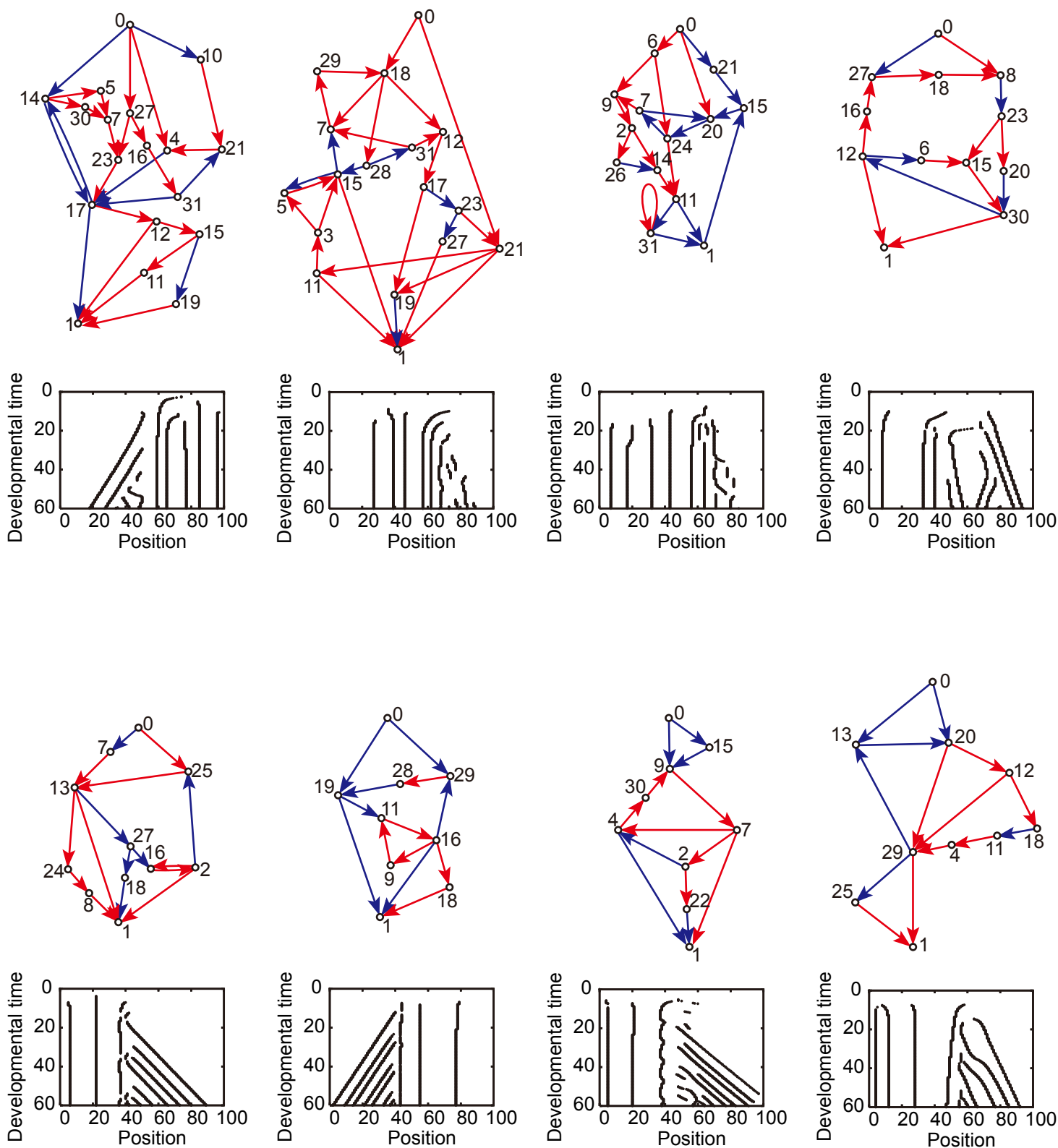

Supplement: Figure S5 — Evolved Networks Show Distinct Network Motifs. Representative examples classified to the three modes are displayed by spatio-temporal expression of gene #1 (lower column) and the corresponding core networks (upper column). (A) Simultaneous gene expression and multiple FFLs in long germ networks. (B) Sequential expression and the presence of a FBL. (C) Combination of simultaneous and sequential expression, and coexistence of FFL and FBL are always observed. (2.14 MB PDF) [file pone.0002772.s007.pdf]

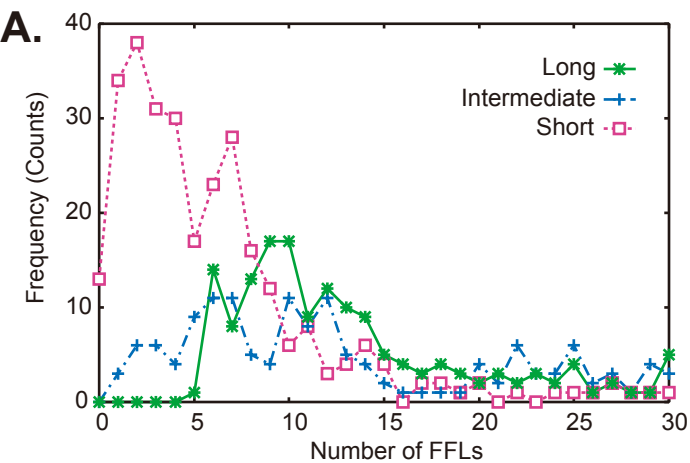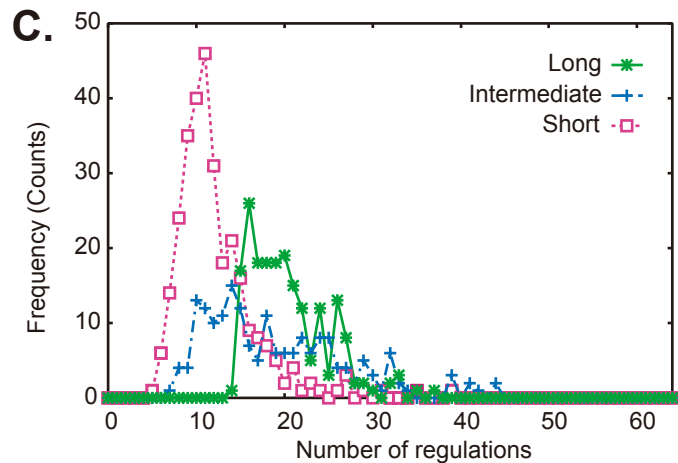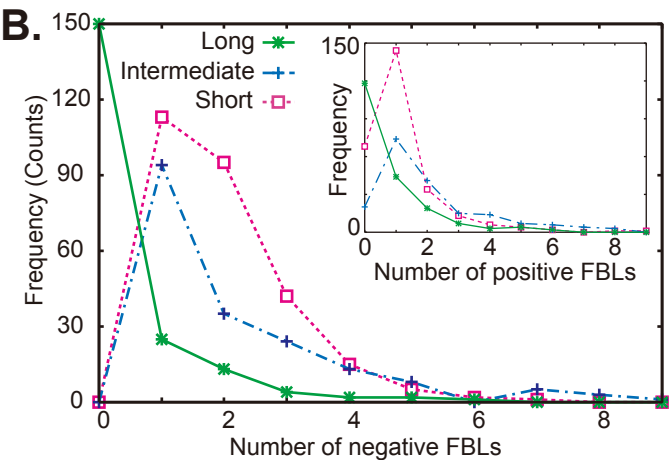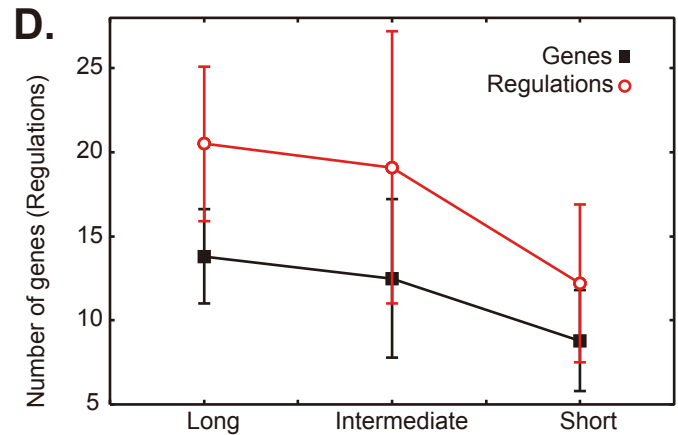

Supplement: Figure S6 — Statistics of Core Network Topologies. (A–C) Frequency distribution of the number of FFLs (A), negative and positive FBLs (B), and regulation, i.e., connections (C) are plotted for core networks of long (green), short (pink), and intermediate (blue) germ modes. The frequency is computed from an ensemble of the core networks used to calculate the distribution in Figure 2D. (D) The average number of genes (black; Fig. 4A) and connections (red; (C)) are plotted for each mode. Error bars indicate the standard deviation. The number of connections as well as the number of genes in the long germ networks is larger than those in the short germ networks. This is because only a single negative FBL is sufficient for the stripe formation in a short germ network to operate, whereas long germ networks require multiple FFLs (Figs. 2D and 3A–B). (0.11 MB PDF) [file pone.0002772.s008.pdf]

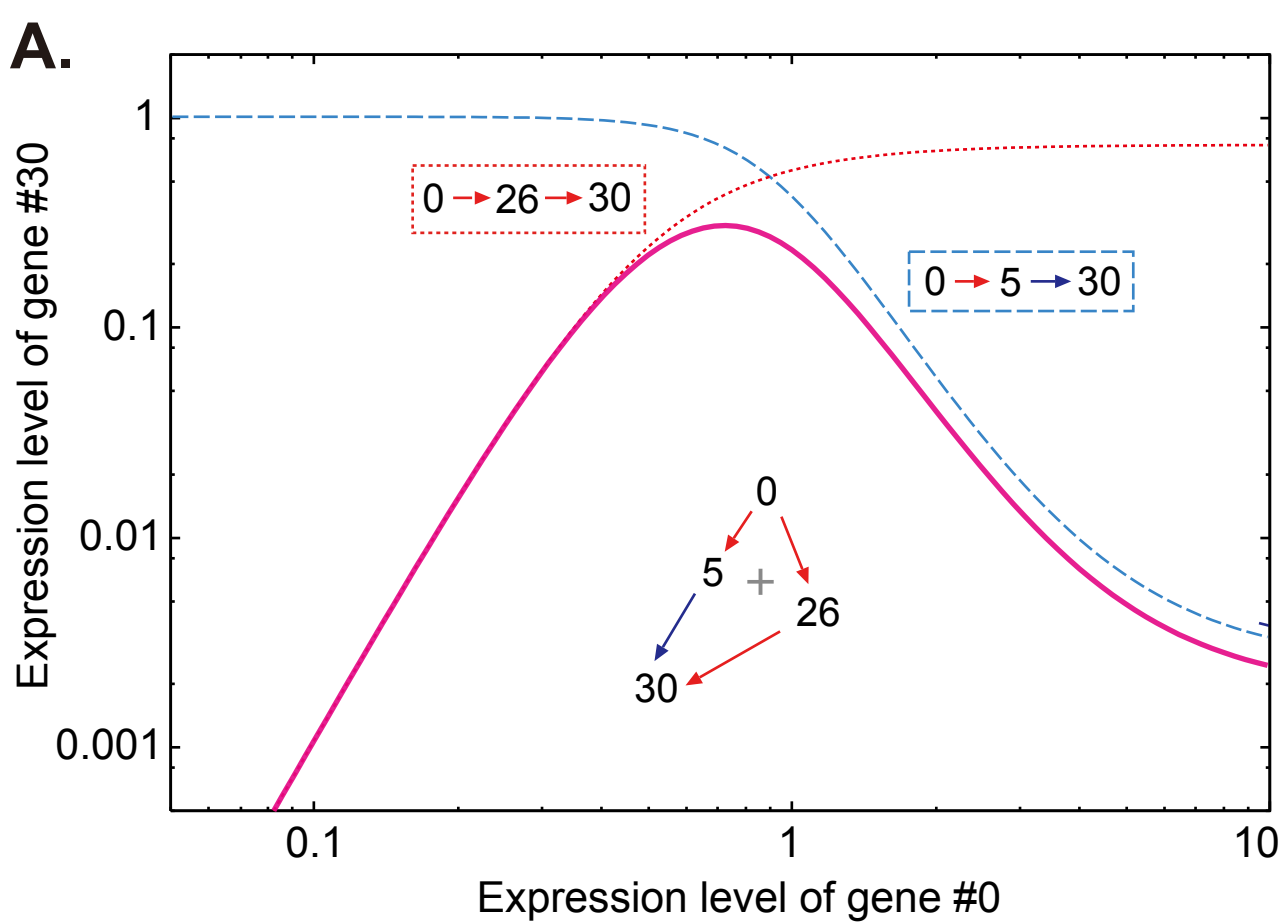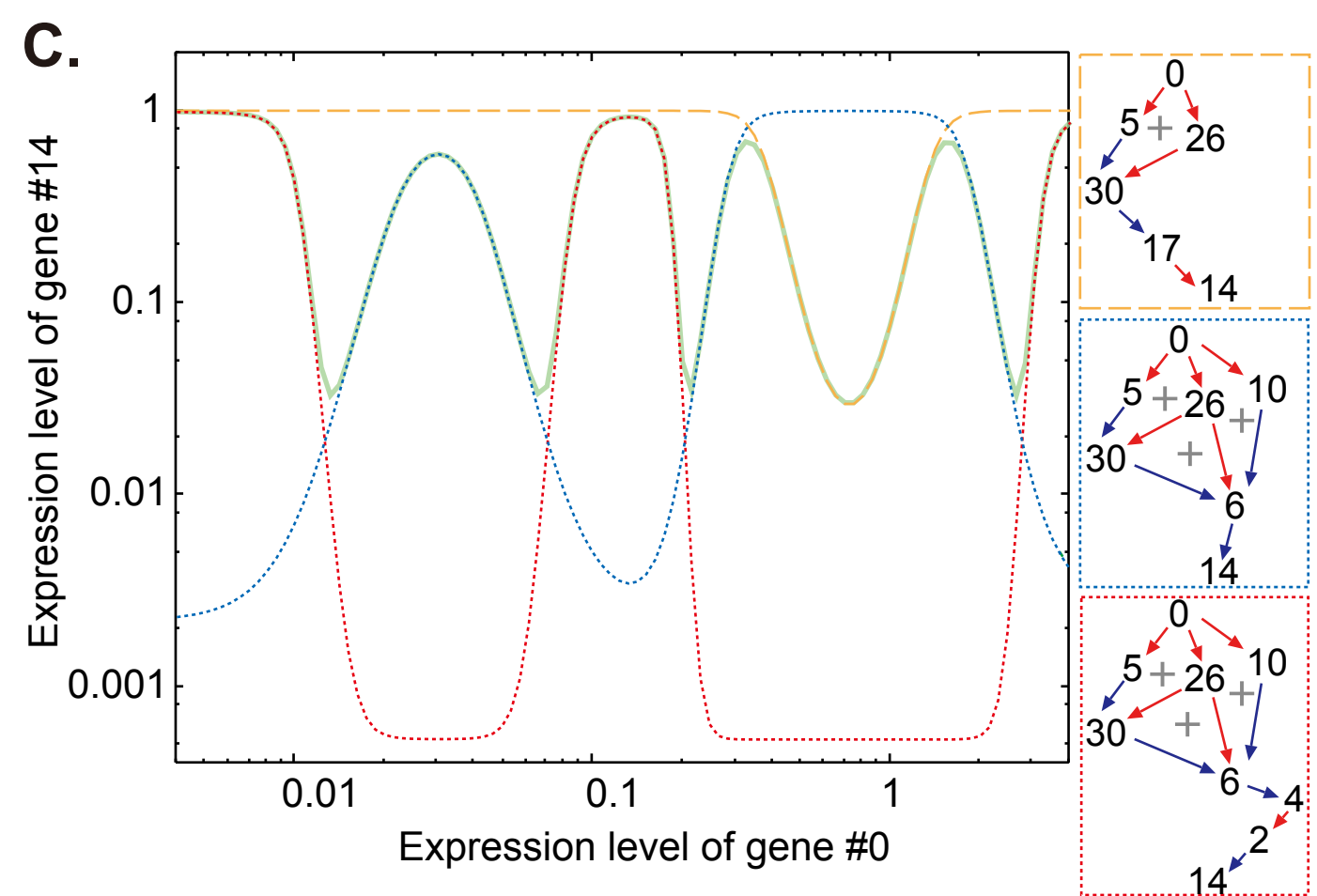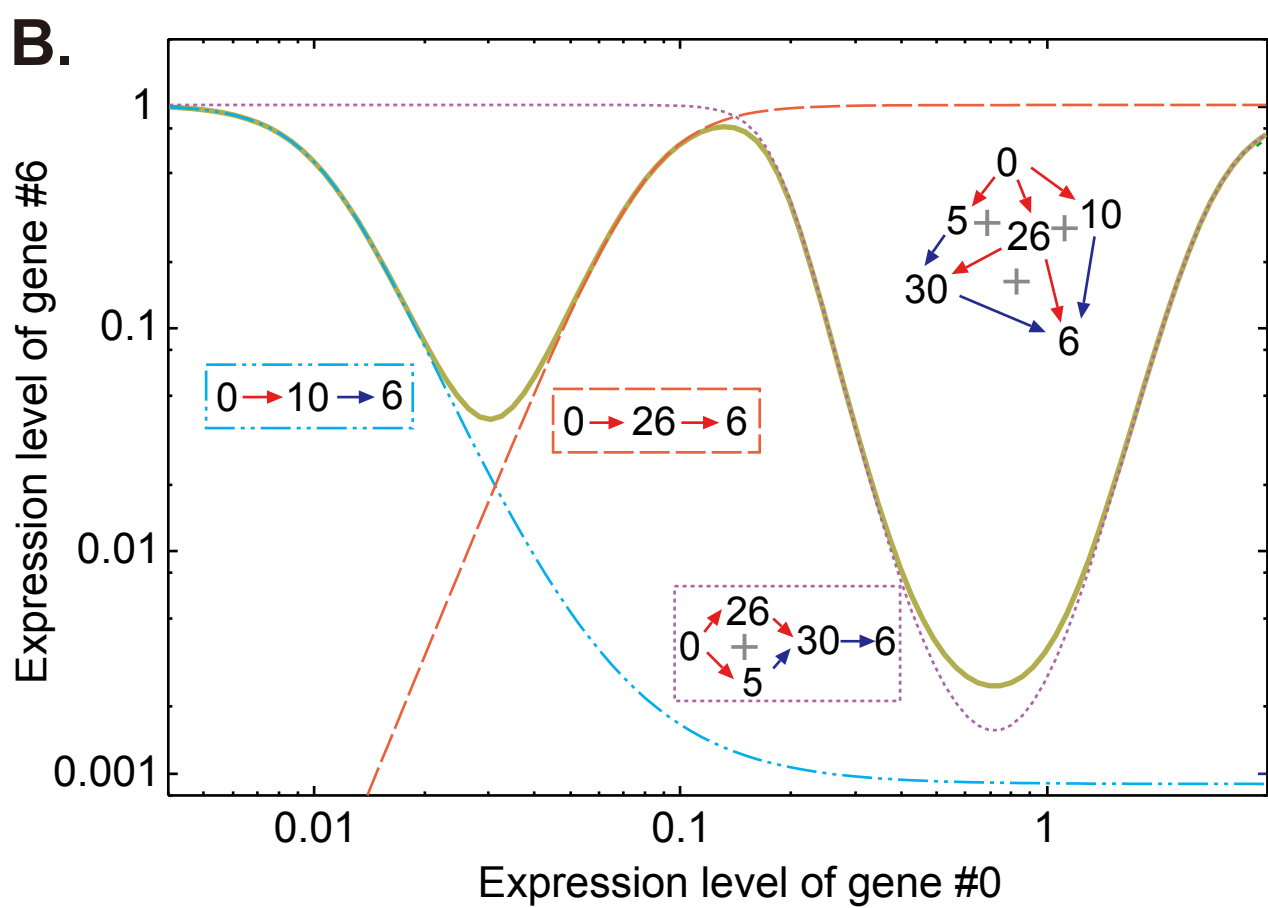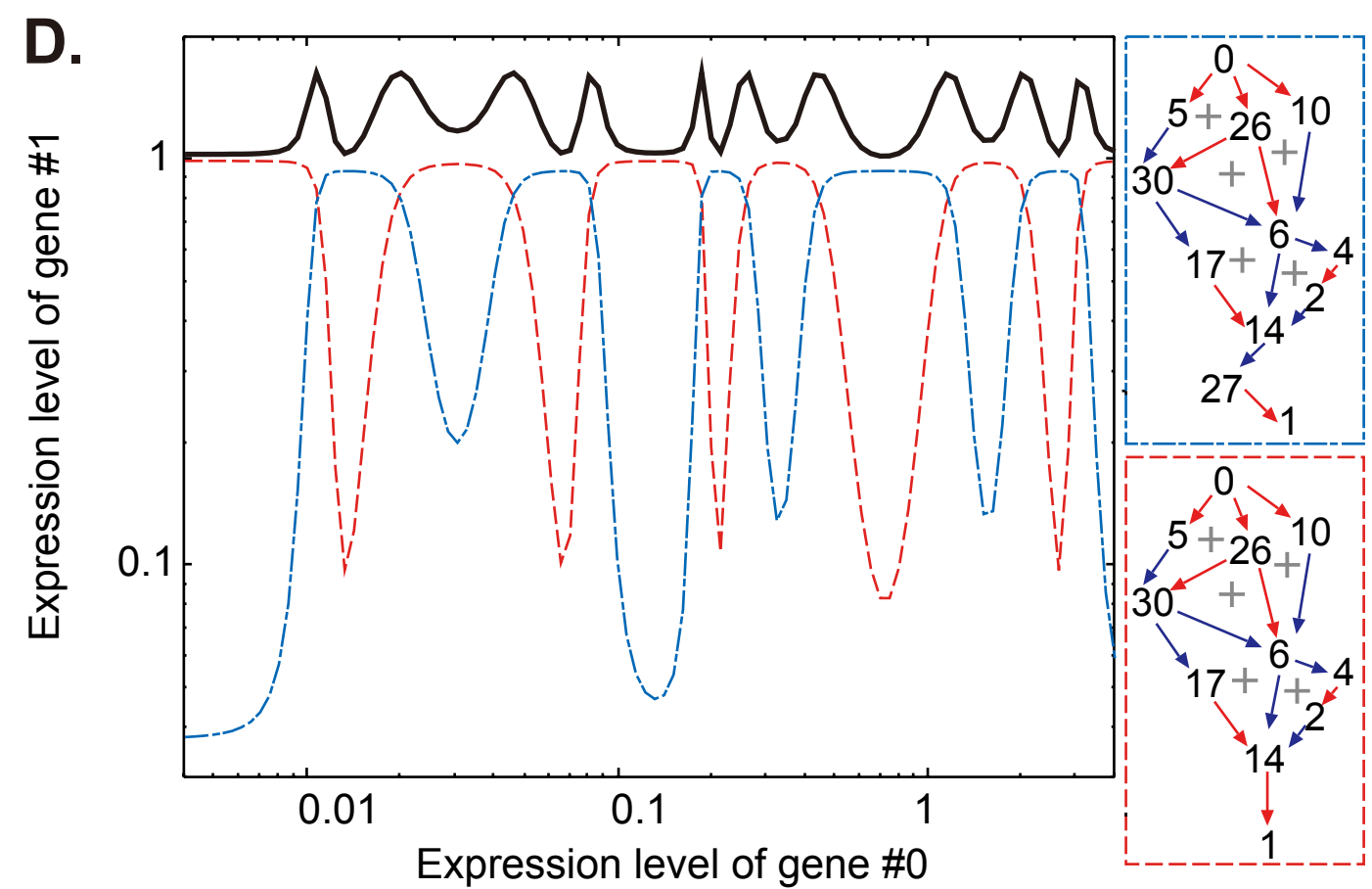

Supplement: Figure S7 — Analyses of Gene Regulation in a Long Germ Network. (A) An isolated FFL, (B,C) FFLs connected in parallel (e.g., marked by+in Fig. 2A) and (D) FFLs connected in series (e.g., * in Fig. 2A). Solid lines represent integrated Gene Regulation Functions (GRFs) for genes #30 (A), #6 (B), #14 (C), and #1 (D) plotted against the expression level of gene #0. The other lines are obtained from decomposed GRF (see Result S1). The colors of lines correspond to the colors of arrows in the decomposed pathways shown in each figure. The integrated GRF forms a local maximum in A, two local minima in B, five local minima in C, and ten local maxima in D. Multiple stripes are generated accordingly (Figs. 1G, S2A and S8). K0→5 = 4, K5→30 = 5×10^−2^, K0→26 = 0.7, K26→30 = 0.6, K0→10 = 6×10^−2^, K10→6 = 3×10^−2^, K30→6 = 1.2×10^−2^, K26→6 = 1.4×10^−2^, K30→17 = 1.5×10^−2^, K17→14 = 1.4×10^−2^, K6→14 = 4.7×10^−2^, K6→4 = 3.6×10^−2^, K4→2 = 2.5×10^−2^, K2→14 = 2.3×10^−2^, K14→1 = 0.1, K14→27 = 0.23, K27→1 = 0.26. (0.16 MB PDF) [file pone.0002772.s009.pdf]

**A.**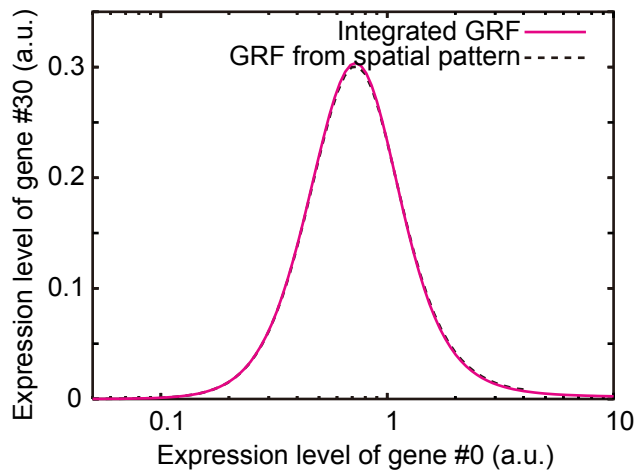**C.**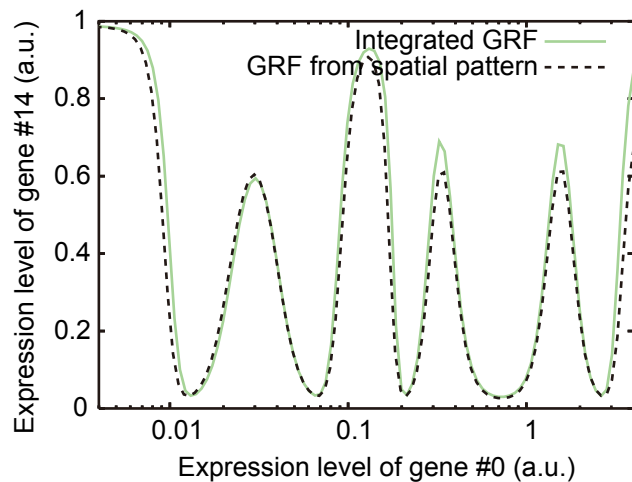**B.**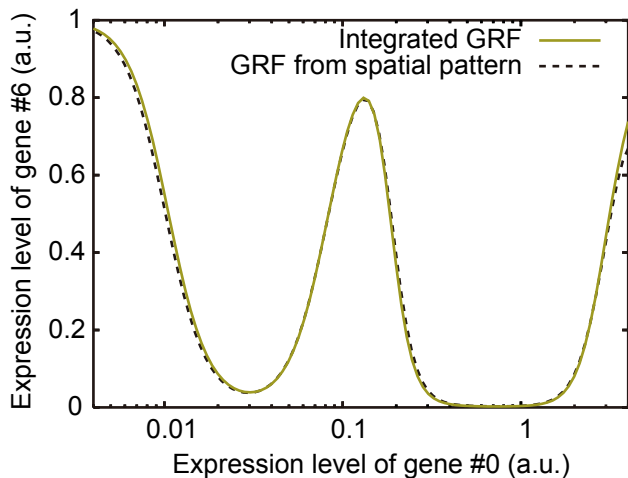**D.**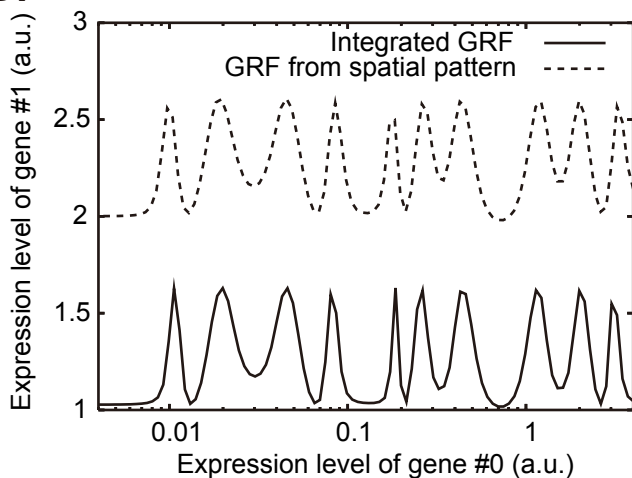

Supplement: Figure S8 — Comparison of GRFs Constructed from a Readout of Spatial Pattern and Integrated GRFs. Dotted lines indicate GRFs of gene (A) #30, (B) #6, (C) #4, and (D) #1 constructed from their spatial patterns shown in Figures 2D and S2A, respectively. Solid lines in A–D indicate corresponding integrated GRFs shown in Figure S7A–D, respectively. They show good agreements with each other. The basal level, however, may be shifted as shown in D. (0.10 MB PDF) [file pone.0002772.s010.pdf]

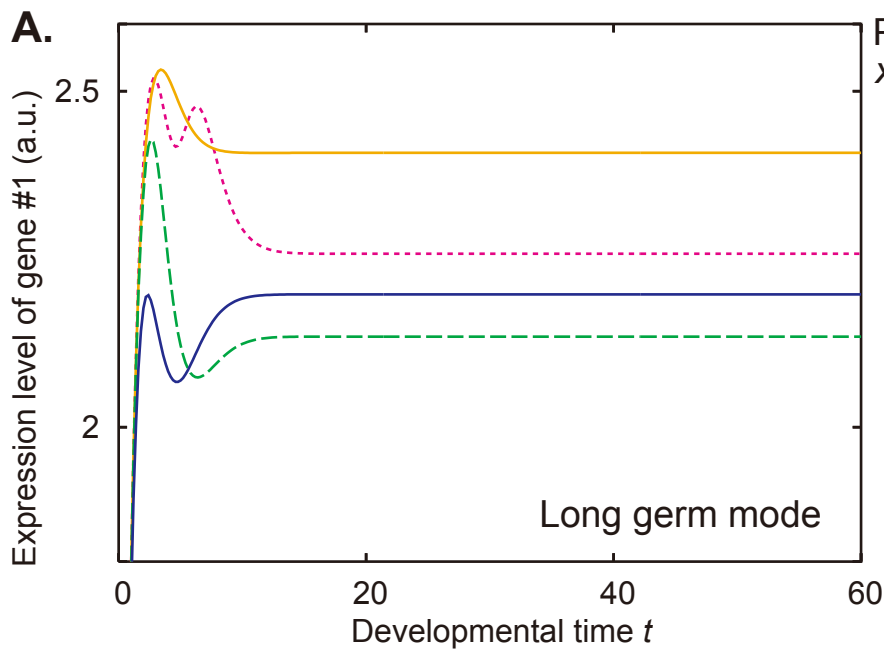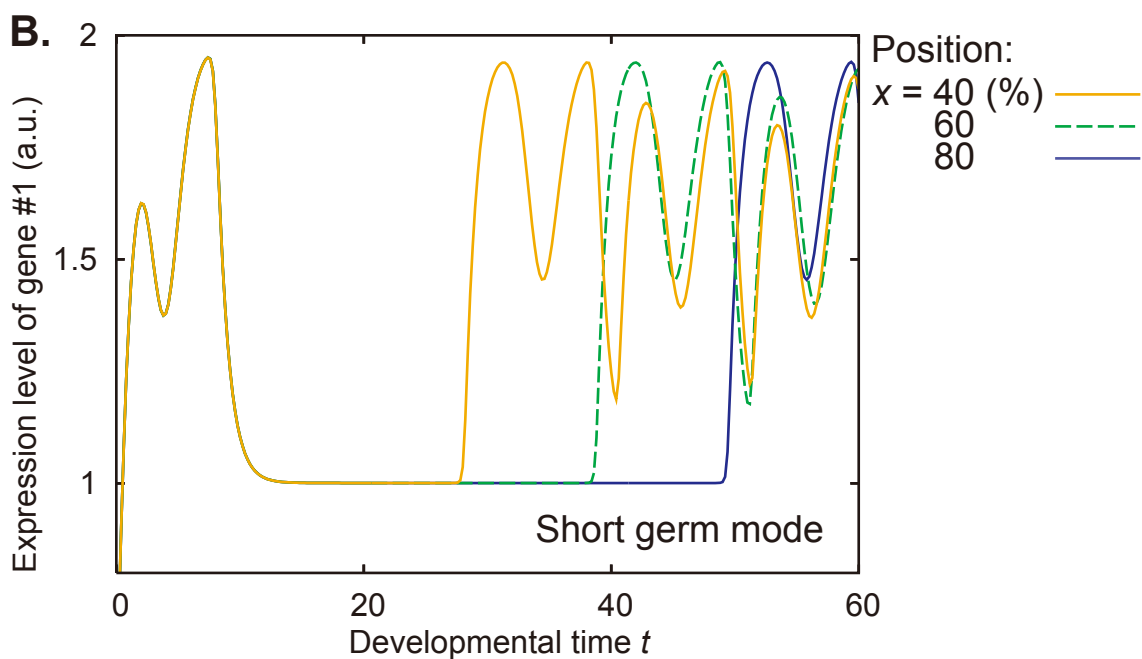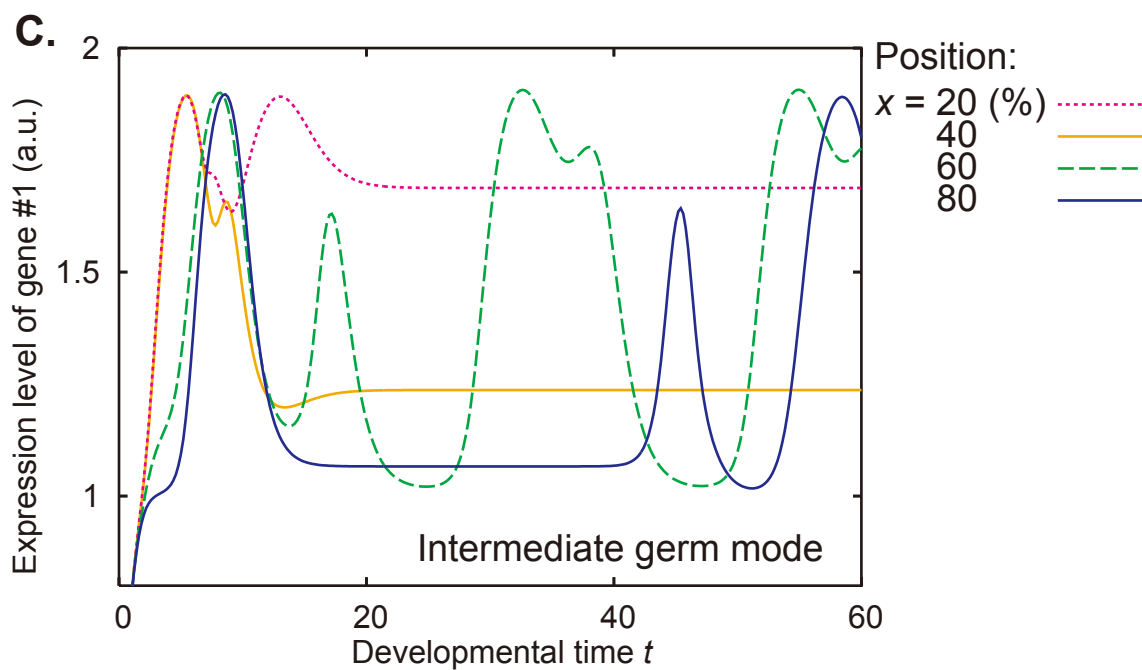

Supplement: Figure S9 — Dynamics of Gene Expression during Development. Expression level of gene #1 is plotted against the developmental time, corresponding to Figure 1D–F, respectively. The spatial positions where the expression level was measured are indicated at the upper right-hand-side corner. (A) After transient expression around t = 8, the levels at all positions are maintained without oscillations. (B) After the transient around t = 8, temporal oscillations appear sequentially, whereby the timing of the appearance is in the order from the left to the right side of an embryo. (C) The level only at x = 60 and 80 shows oscillation in a sequential order. (0.12 MB PDF) [file pone.0002772.s011.pdf]

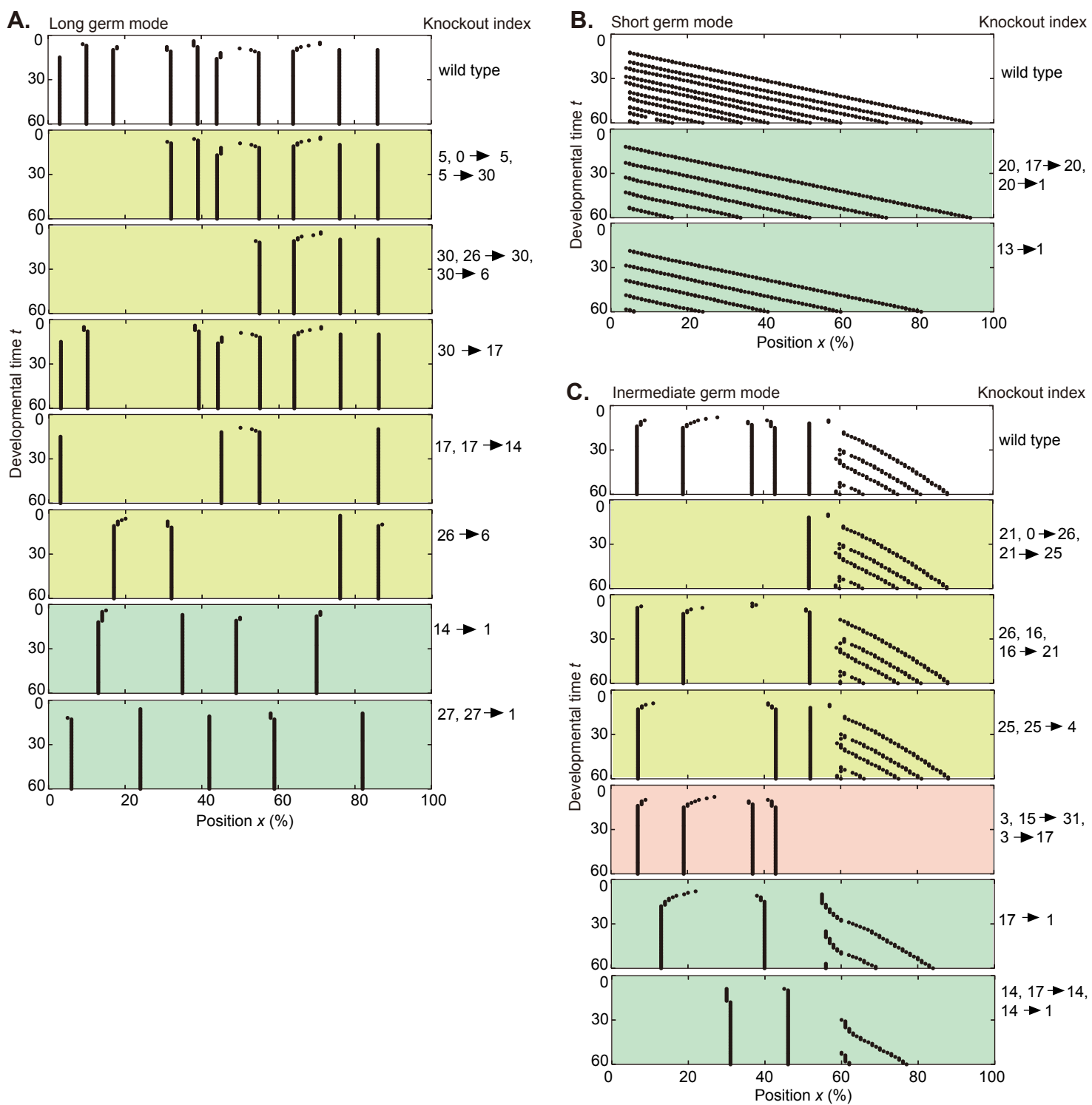

Supplement: Figure S10 — Developmental Time-course of Mutated Networks. Panels show the expression dynamics of gene #1 organized by a mutant network denoted on the right side. Local maxima of the expression level of gene #1 are plotted at each developmental time t. They are in the same conditions as in Figure 3A–C, respectively, except for the cases where the stripes are extinguished throughout the development (the lowest panel in Fig. 3A and pink panels in Fig. 3B). (0.38 MB PDF) [file pone.0002772.s012.pdf]

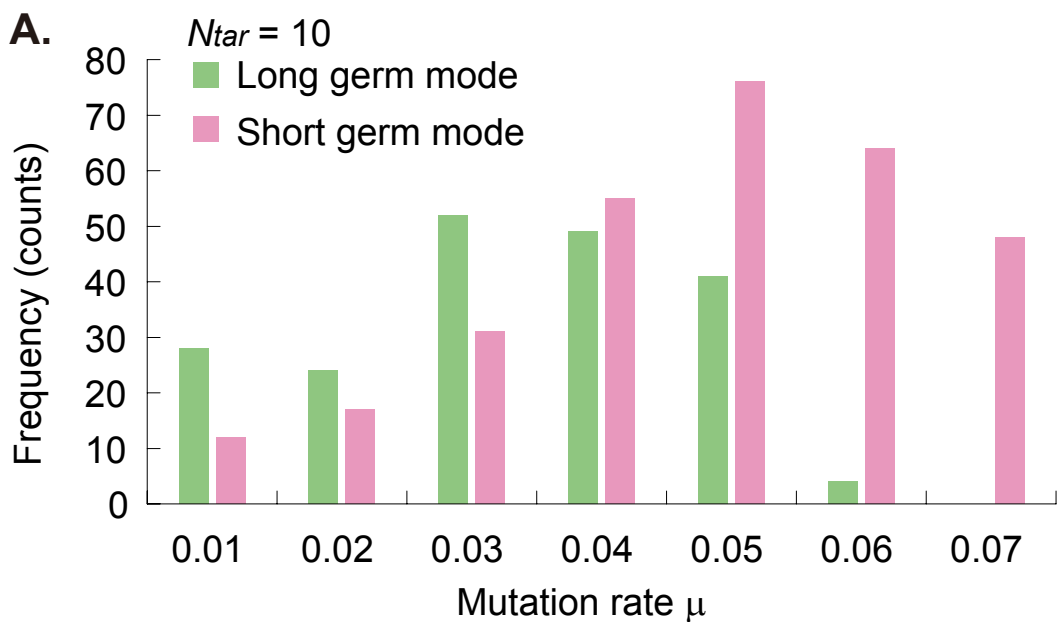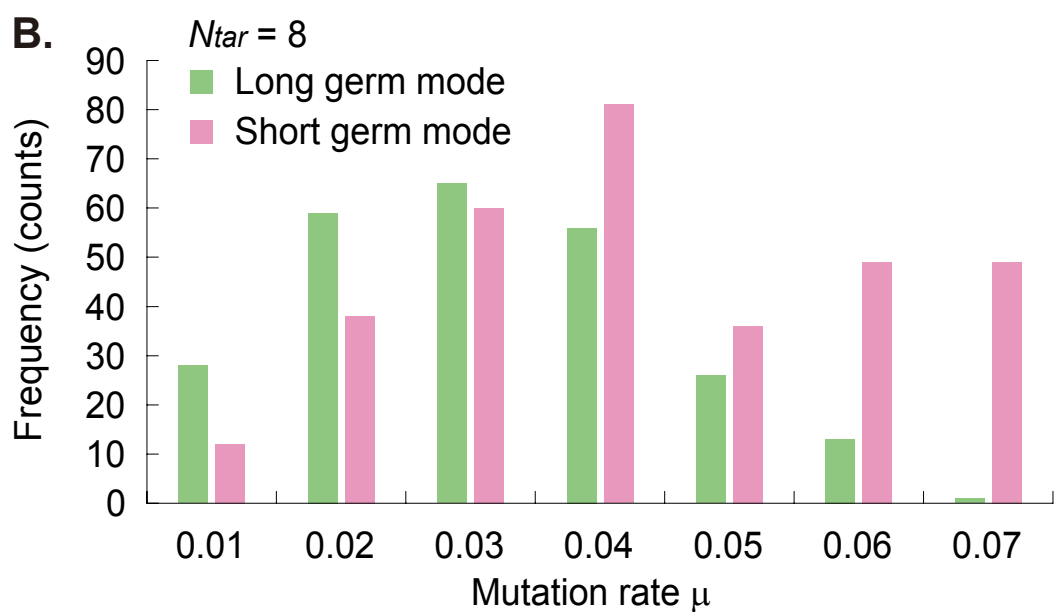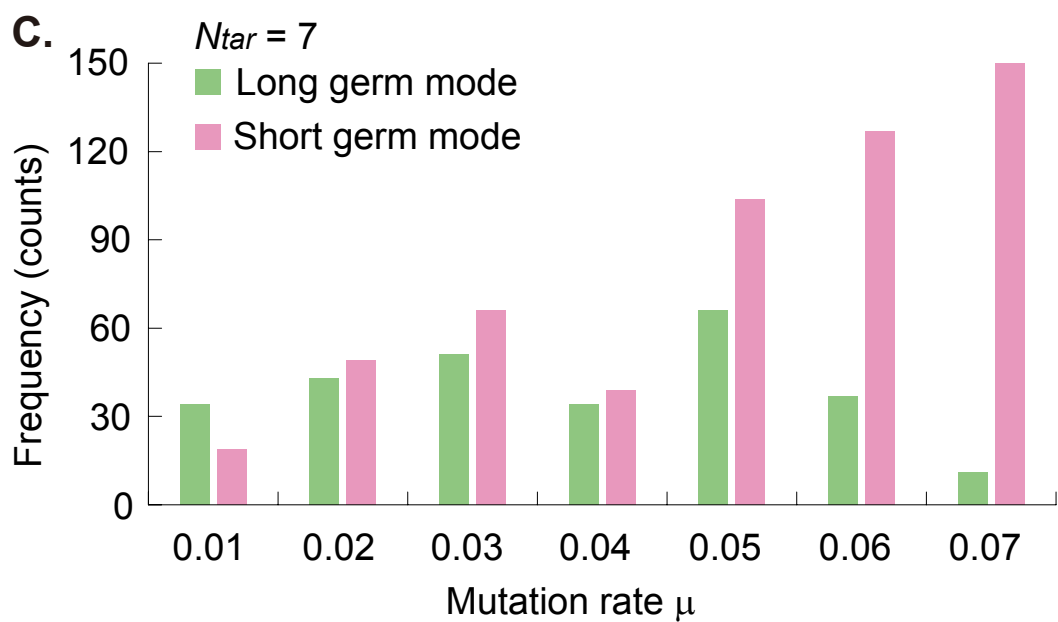

Supplement: Figure S11 — Short Germ Modes Appear More Frequently in Evolution When the Mutation Rate is High. Approximately four hundred independent evolutionary trials are examined for a different mutation rate μ and target number Ntar = 10 (A), 8 (B), and 7 (C), appearance of long and short germ modes are counted respectively at 2000th evolutionary generation. The frequencies are plotted against μ. The ratio of the frequencies, i.e., the frequency of long germ modes divided by that of the short germ modes, is shown in Fig. 4C. (0.11 MB PDF) [file pone.0002772.s013.pdf]

## A. Long germ mode

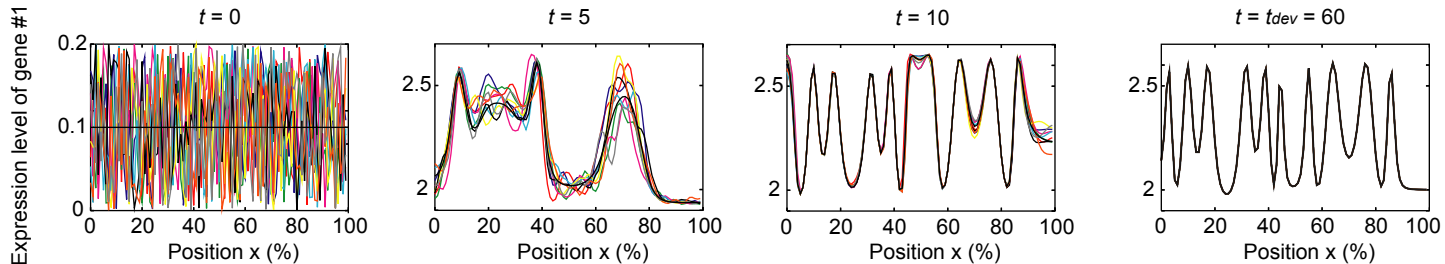

## B. Short germ mode

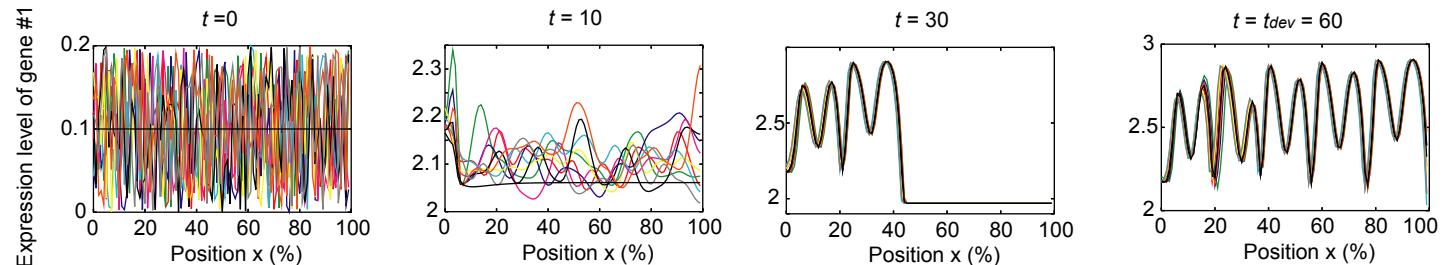

## C. Intermediate germ mode

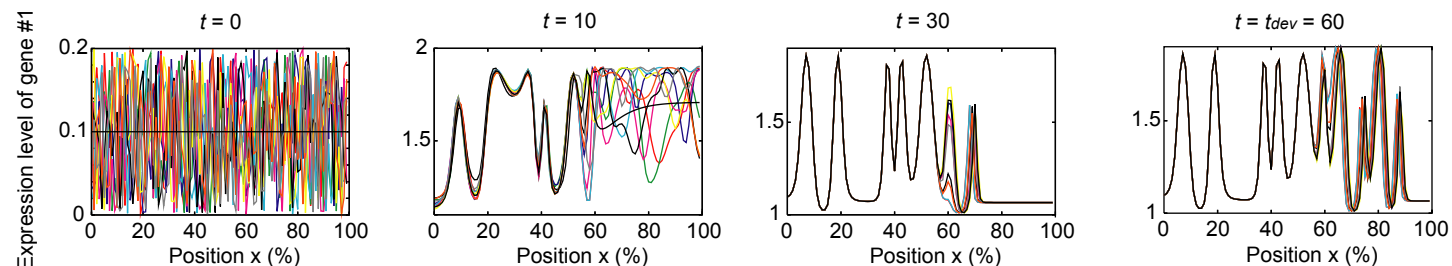

Supplement: Figure S12 — Developmental Robustness of Evolved Networks. Robustness of gene expression pattern against perturbations to the initial conditions (left figure in A–C) was studied. The expression patterns of gene #1 at several time points are shown. Black lines indicate the unperturbed development shown in Figure 1D–F. The variations of the patterns arising from different initial conditions are filtered out during development in all three modes. Simultaneous stripe formation (A and anterior region of B) shows a relatively smaller variation than the sequential one (posterior region of B and C). (0.17 MB PDF) [file pone.0002772.s014.pdf]

**A**

CV of striped pattern

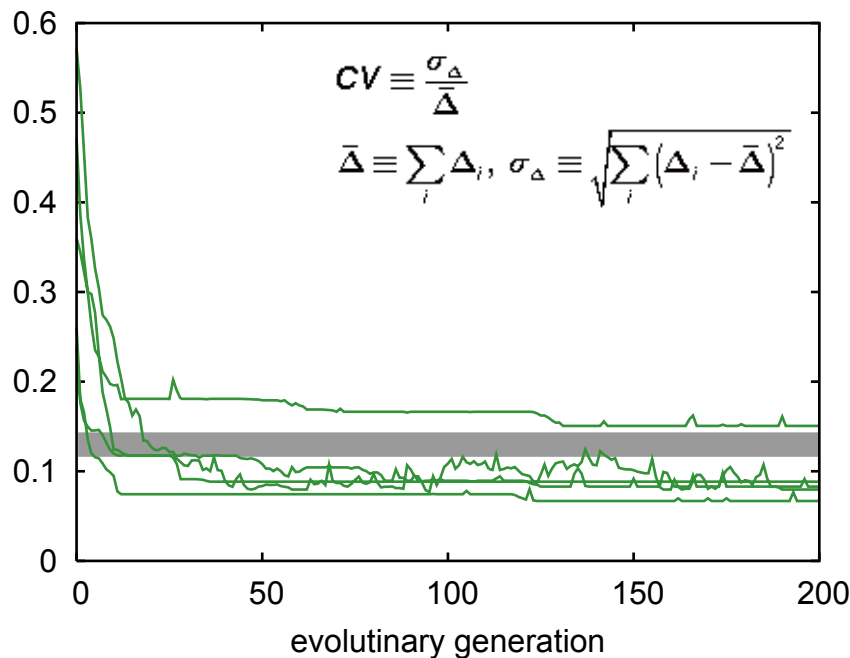**C**

Fraction of networks

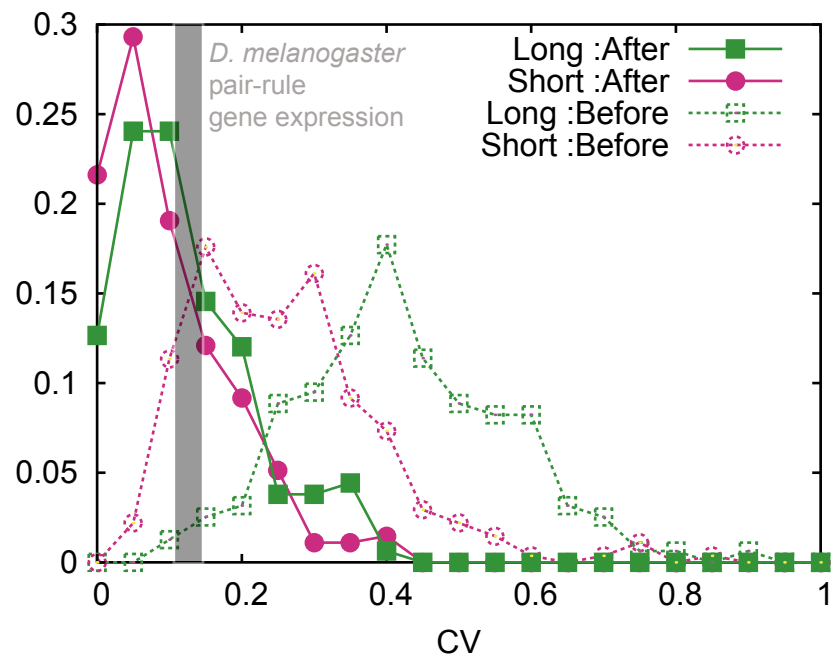**B**

Expression level (a.u.)

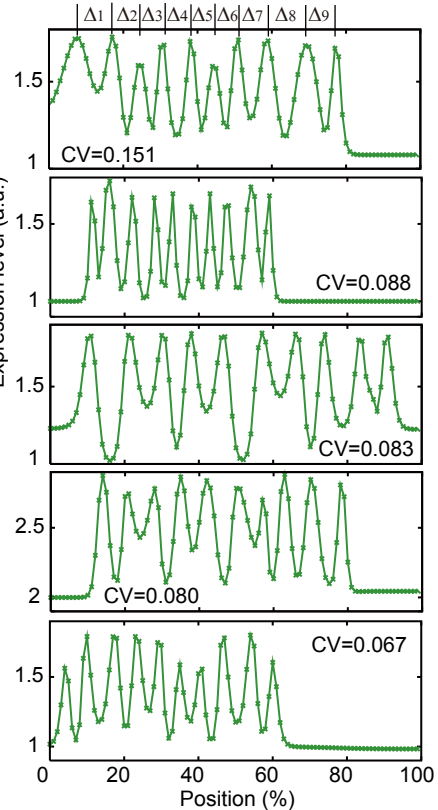**D**

Expression level (a.u.)

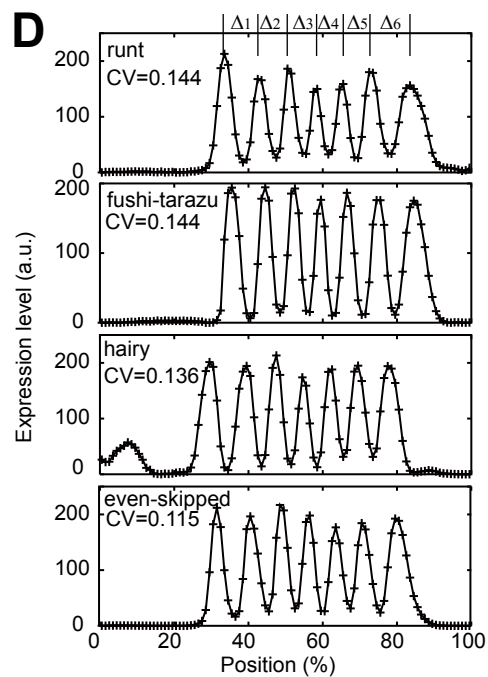

Supplement: Figure S14 — Evolutionary Parameter Tuning of FFLs Leads to More Regular Stripes. (A)–(C) Evolution of Coefficient of Variation (CV) of peak-to-peak distance in the striped pattern (See Methods S1). Average of the CV among the selected embryos is plotted against generation steps in A. Five representative trials each of which adopts a different network connection yielding long germ mode development. Striped pattern after each evolution is shown in B. In C, the evolution is applied independently to the all networks within long (green) and short (pink) germ modes. Dotted and solid lines indicate frequency of CV before and after the evolution, respectively. Shaded region in A and C indicates CV calculated from striped pattern of D. melanogaster in D. The CVs in long germ modes decrease to the same level as those in short germ modes as well as in D. melanogaster. For 60% of the long germ networks, the CV after the evolution is not over that in D. melanogaster. (D) The pair-rule gene expression patterns of D. melanogaster (Methods S1). Seven stripes with larger expression level were adopted to calculate CV and the other smaller peaks were not adopted. (0.17 MB PDF) [file pone.0002772.s016.pdf]
