# Supplementary material for: Network Evolution of Body Plans
Source: PLoS One. 2008 Jul 23;3(7):e2772. doi: 10.1371/journal.pone.0002772 (PMC2464711; doi:10.1371/journal.pone.0002772)
Supplement: Figure S13 — Network Modules Included in Core Networks. Decomposition of networks into modules are demonstrated. The networks are shown in Figure 2A–C. (A) There are five FFLs connected in parallel and a FFL connected in series. (B) There are a FFL connected in series, a negative FBL and a positive FBL. (C) There are four FFLs connected in parallel, a FFL connected in series, a negative FBL and positive FBL. By systematically decomposing the other evolved networks into the modules, statistics in Figures 2D and S7A–B were measured. (0.10 MB PDF) [file pone.0002772.s015.pdf]

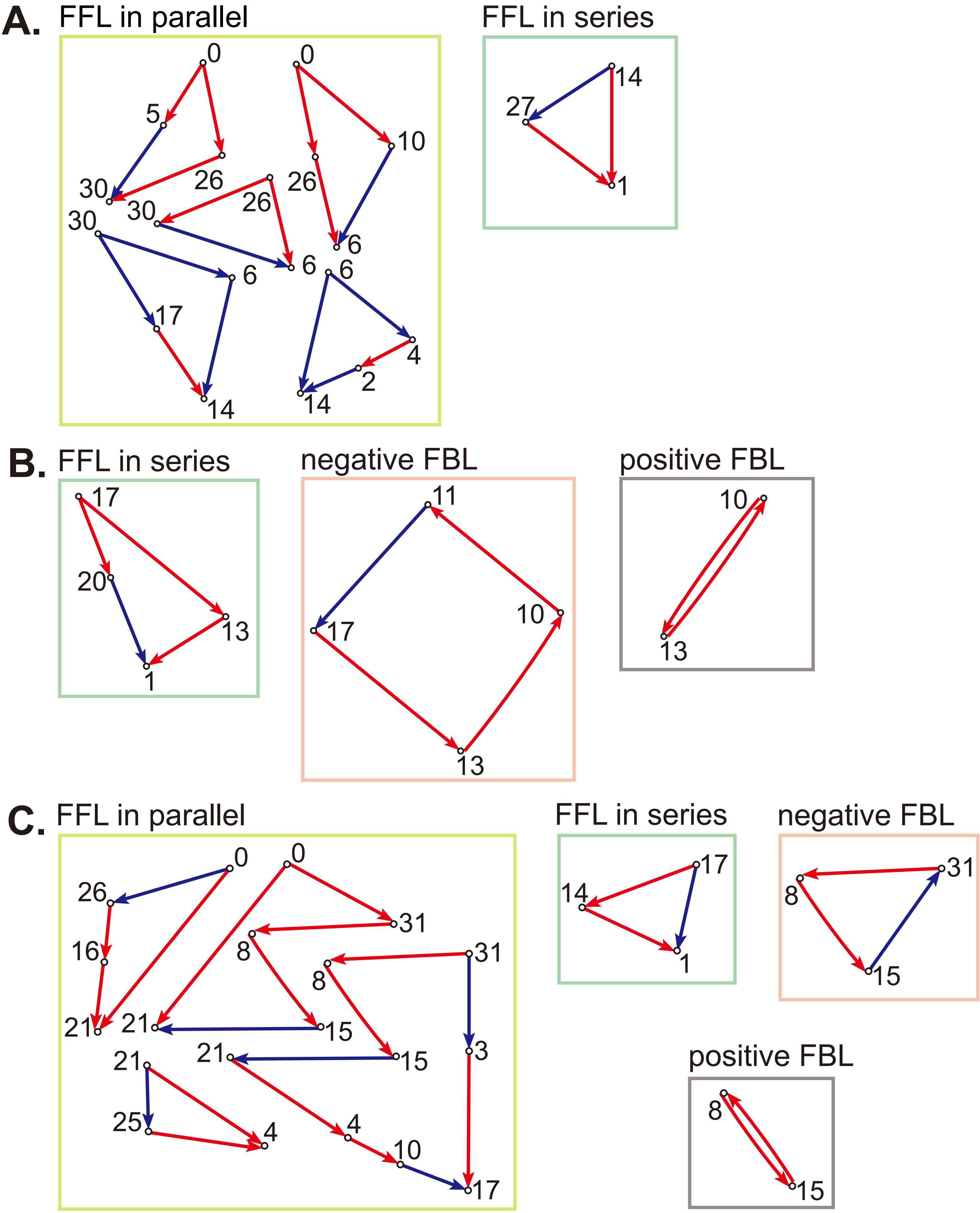
A.
FFL in parallel
FFL in series
B.
FFL in series
negative FBL
positive FBL
C.
FFL in parallel
FFL in series
negative FBL
positive FBL
